# PBAE nanoparticle-mediated delivery of ASCL1 and NGN2 genes for astroglia-to-neuron reprogramming to remodel glial scar for spinal cord injury repair

**DOI:** 10.1186/s13287-026-05159-2

**Published:** 2026-07-04

**Authors:** Jianbin Guo, Lin Li, Zijian Liu, Shihao Yuan, Xiaoyu Ma, Dandan Zhang, Peng Deng, Jinchao Wang, Bo Chen, Jing An, Junping Li, Quanrui Ma, Hao Yang

**Affiliations:** 1 Department of Joint Surgery, Hong Hui Hospital, Xi’an Jiaotong University, Xi’an, 710054 China; 2 Translational Medicine Center, Hong Hui Hospital, Xi’an Jiaotong University, Xi’an, 710054 China; 3 Department of Anatomy, Histology and Embryology, School of Basic Medicine, Ningxia Medical University, Yinchuan, Ningxia Hui Autonomous Region 750004 China

**Keywords:** Direct neuronal reprogramming, Glial scar, Poly(beta-amino ester), Gene delivery, Spinal cord regeneration

## Abstract

**Background:**

Irreversible loss of neuronal cells elicited by neurotraumatic injuries or neurodegenerative disorders is particularly devastating due to the limited regenerative capacity of the central nervous system (CNS). Cell reprogramming-based therapies have emerged as promising therapeutic avenues for neuronal replenishment. However, their therapeutic potential in neural regeneration still faces formidable challenges, including risks of viral vector gene delivery, potential damage from cell transplantation, and significant glial scar (GS) formation following CNS injury. Therefore, developing an optimal approach that simultaneously replaces lost neurons and overcomes these persistent obstacles is crucial for neural regeneration and functional recovery.

**Methods:**

We engineered a non-viral gene delivery platform using biodegradable poly(β-amino ester) (PBAE) nanoparticles (NPs) to effectively co-deliver plasmids encoding proneural transcription factors ASCL1 and NGN2 directly to astroglia (ATG) within GS region, in combination with neural induction. The biochemical and physiological properties of reprogrammed ATGs were characterized both in vivo and in vitro. The therapeutic potential of PBAE-A/N delivery was assessed in spinal cord injury (SCI) animal models through behavioral evaluations. Finally, the molecular mechanisms underlying ASCL1/NGN2-mediated ATG-to-neuron reprogramming were investigated.

**Results:**

PBAE-mediated delivery of ASCL1/NGN2 plasmids effectively reprogrammed resident ATGs within GSs into functional neurons, as evidenced by the acquisition of neuronal morphology and biochemical phenotype (neuronal marker expression), loss of ATG characteristics, scar remodeling, and functionality indistinguishable from those of genuine neurons, including specialized calcium signaling, synaptic activity, and action potential firing. Critically, local administration of PBAE-ASCL1/NGN2 NPs into the GS region of the injured spinal cord significantly ameliorated neurological deficits. Mechanistically, this reprogramming event likely involved the modulation of downstream targeting signaling mediated by Cend1, RanBPM, and Dyrk1, along with crosstalk with the Notch1/Cyclin D1 axis.

**Conclusions:**

This study demonstrates that PBAE-mediated ASCL1/NGN2 delivery enables in situ reprogramming of ATG into functional neurons while actively dissolving GSs, thereby addressing both neuronal loss and GS barriers in CNS repair. The identified Cend1/RanBPM/Dyrk1 signaling and its crosstalk with Notch1/Cyclin D1 axis provide mechanistic insights into the events. Collectively, this work presents a novel therapeutic alternative for CNS repair and neurodegeneration by simultaneously replacing lost neurons and eliminating endogenous GSs through in situ cell reprogramming.

**Graphical Abstract:**

**Figure:**
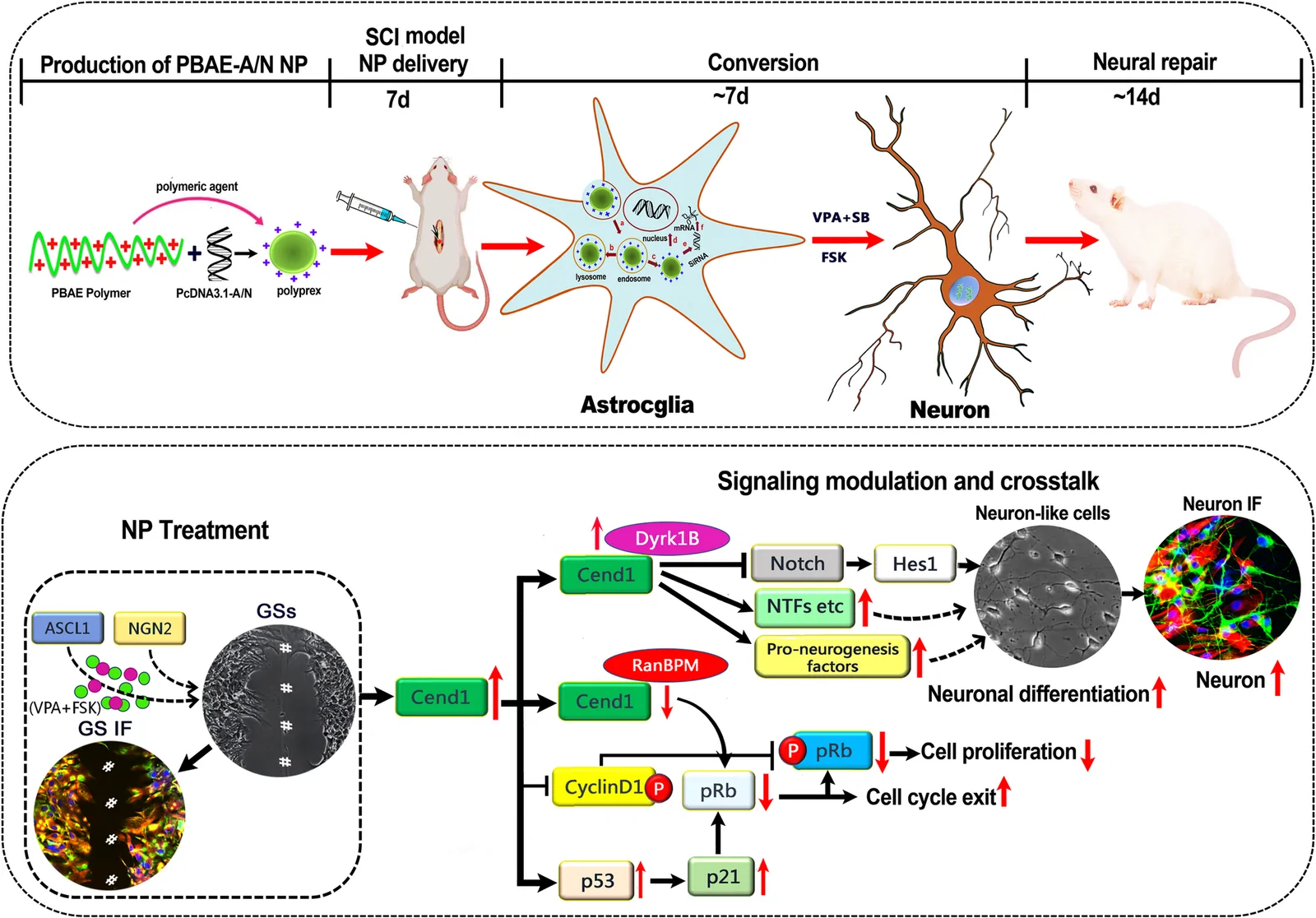
Schematic illustration of astroglia-to-neuron reprogramming and glial scar remodeling for spinal cord repair, along with proposed underlying mechanisms. Synthesis and targeted delivery of PBAE nanoparticles encapsulating ASCL1/NGN2 into SCI lesion region, enabling in situ conversion of astroglia within the glial scar into functional neurons and facilitating structural and functional recovery (upper panel). (B) Proposed molecular mechanism by which PBAE NP-mediated ectopic expression of ASCL1 and NGN2 orchestrates the reprogramming resident astroglia into neuronal lineages (lower panel). IF: immunoflourescence; NP: nanoparticle; GS: glial scar; NTFs: Neurotrophic factors

**Supplementary Information:**

The online version contains supplementary material available at https://doi.org/10.1186/s13287-026-05159-2.

## Background

The irreversible degeneration of neural tissue caused by degenerative diseases or traumatic injuries is particularly devastating because of the limited regenerative capacity of the central nervous system (CNS) [1–3]. To date, no definitive curative treatments exist, and current therapeutic strategies, including surgery and medication, have extremely limited efficacy and cause numerous complications [4–7]. Consequently, crucial and long-standing questions are whether the destroyed neural tissue can be effectively replaced and which approaches are effective for restoring neurological function. Fortunately, stem cell-based therapies have emerged as among the most promising treatment options for counteracting irreversible neural cell loss [8–12]. Although stem cell transplantation holds considerable promise for facilitating CNS repair, such as by replacing insulted cells, reducing neuroinflammation, promoting axonal sprouting, and restoring neuronal circuitry [13–17], its clinical translation for neural regeneration still faces formidable challenges [18]. These challenges include very limited cell sources, ethical controversies, immunorejection, and inflammatory responses [19–23]. Therefore, identifying a desired stem cell reservoir capable of generating functional neurons while circumventing the abovementioned drawbacks is imperative.

Recently, a growing number of studies have shown that astroglial cells (ATGs) can be converted into multipotent neural stem cells (NSCs), lineage-restricted neural progenitors, and even specific neuronal subtypes through the forced expression of defined transcription factors (TFs) or specific interventions, such as small molecule induction, targeted signaling modulation, and other combined stimulatory approaches [12, 16, 24, 25]. Notably, direct ATG-to-NSC reprogramming can be accomplished without neural cell lineage switching or the use of invasive grafts [12, 16, 25, 26]. As a result, direct neuronal reprogramming of ATGs represents a promising strategy for CNS repair following traumatic injury and degenerative diseases [12, 27–30]. Nonetheless, cell replacement therapy based on direct reprogramming of ATGs into neuronal cells still faces enormous challenges in terms of achieving efficient neural regeneration and functional recovery, largely due to the inhospitable environment resulting from the GS [31]. In general, any forms of CNS insults, including various severe traumatic brain and spinal cord injuries, stroke, and neurodegenerative disorders, usually trigger glial scar (GS) formation [32–34]. Although the GS initially plays a protective role by shielding the noninjured tissue from injury during the early period, curtailing the spread of inflammation, and stabilizing the extracellular environment during the acute phase [35–40], it eventually becomes detrimental. It forms a physical barrier for neurite outgrowth and produces redox reactants and inflammatory mediators that further aggravate tissue damage, ultimately leading to persistent neurological dysfunction. Accordingly, a critical strategy for removing the GS and fostering a more permissive environment is required for CNS function recovery.

In this study, we present a highly efficient induction system for directly reprogramming of ATGs within the GS into neurons with characteristic neuronal morphology and biochemical phenotypes by delivering biodegraded PBAE NPs carrying the proneural TFs ASCL1 and NGN2. Moreover, we show that the biodegradable PBAE NPs function as a nonviral vector with high transfection efficiency and induce the reprogramming of GS cells into induced neurons (iNeurons) through the intrinsic biomaterial-mediated expression of ASCL1 and NGN2. This process ultimately leads to GS remodeling and enables axonal outgrowth. Strikingly, the cationic PBAE polymer class designed for nonviral gene delivery is safe owing to its nonimmunogenicity and hydrolytic degradation and possesses a high capacity for DNA cargo [41–43]. More importantly, our approach enables the efficient coloading of TF plasmids onto PBAE NPs, resulting in superior cotransfection performance. Therefore, this strategy effectively overcomes the drawbacks of conventional cotransfection methods that rely on single vectors carrying multiple genes. Furthermore, the appropriate size of PBAE NPs for carrying two TF plasmids facilitates easier penetration into GS cells, thereby improving cotransfection efficiency. Using this strategy, we achieved three important breakthroughs: (i) synchronous epigenetic modifications with two TFs, thus circumventing partial or incomplete intermediate states; (ii) in situ reprogramming of GS cells to avoid neural cell lineage switching and exogenous cell transplantation; (iii) reduced intrinsic risks with viral vectors and simplified procedural complexity. Of significance, the local administration of TF-loaded PBAE NPs into the GS region resulted in substantial GS remodeling and promoted functional recovery in an animal injury model. Mechanistic studies revealed that Cend1/RanBPM/Dyrk1-targeted downstream cascades in collaboration with Notch1/Cyclin D1 signaling to contribute to this intricate cellular reprogramming and GS resolution. These findings provide critical insights into the design of a PBAE-based NP system combined with TFs for effective treatment of CNS injury and neurodegenerative disorder through in situ glial cell-based replacement.

## Materials and Methods

### Culture of astroglial cells and astroglial scar model

All animal experiments were approved by the Institutional Animal Care and Use Committee (IACUC) of Xi’an Jiaotong University (Approval No. 202003058) and were conducted in accordance with the ARRIVE guidelines and relevant regulations. All efforts were made to minimize animal suffering. The approved project title was “PBAE nanoparticle-mediated transduction of proneural transcription factors ASCL1 and NGN2 to dissolve astroglial scar for spinal cord regeneration." Rats were obtained at Xi’an Jiaotong University and housed under specific pathogen-free conditions with a 12 h light/dark cycle and free access to food and water. Primary cultures of ATGs were prepared from the cerebral cortex of embryonic day 15 Sprague–Dawley (SD) rats following a previously described method [44]. Briefly, pregnant rats were deeply anesthetized with an intraperitoneal injection of sodium pentobarbital (40 mg/kg). Following confirmation of non-responsiveness to noxious stimuli, euthanasia was performed by cervical dislocation for primary isolation. Concomitantly, embryos were dissected from the uterus, and cortical tissues were isolated and enzymatically dissociated to obtain a single cell suspension. The cells were then seeded into PLL-coated plastic flasks (60 mm in diameter) at a density of 1 × 10^6^ cells/cm^2^. After in vitro incubation for about 7 to 10 days, the cells were subcultured into new 6-well plates using trypsinization. When the cell confluency reached approximately 95%, the cultures were maintained for subsequent experiments.

An astroglial wound model was developed according to the method described in our previous study [45]. After the ATG monolayers were wounded by making longitudinal and latitudinal scratches with an iris blade. The scratched cultures were then maintained *in vitr*o for 1, 3, 5, and 7 days, respectively, before being used in the following experiments.

### PBAE synthesis and characterization

Poly(β-amino ester) (PBAE) was synthesized via a two-step Michael addition reaction according to previously reported methods [46, 47]. Briefly, 1,4-butanediol diacrylate and 4-amino-1-butanol were reacted at molar ratios of 1.1:1 and 1.2:1 for 24 h at 90 °C under continuous stirring overnight to yield PBAE polymers with molecular weights of approximately 6 kDa and 4 kDa, respectively. The resulting polymers were precipitated in cold diethyl ether, washed, vacuum-dried, and subsequently lyophilized. Molecular weight (MW) was determined using nuclear magnetic resonance (NMR) and further characterized by gel permeation chromatography (GPC). Concomitantly, the base polymers were dissolved in anhydrous tetrahydrofuran (THF) at 100 mg/ml and added to end-capping E monomers (0.5 M in THF) at a volume ratio of 3:2. This mixture was allowed to react at room temperature with stirring for 1 h. Subsequently, the PBAE polymers were washed twice with diethyl ether to remove unreacted monomers and oligomers. Residual solvents were evaporated in a vacuum desiccator for 2 days. Finally, all polymer samples were dissolved in DMSO at 100 mg/ml and stored at − 20 °C until further use.

^1^H NMR spectroscopy (Bruker 500 MHz) was conducted in CDCl3 NMR to characterize polymer structure, and the data analysis was performed using TopSpin 3.5 software (Billerica, MA, USA). Gel permeation chromatography (Waters, Milford, MA) measurements were carried out to measure polymer molecular weight and polydispersity. Polymer samples were dissolved in a mixture of BHT-stabilized tetrahydrofuran with 5% DMSO and 1% piperidine, filtered through a 0.2 μm PTFE filter and measured against linear polystyrene standards.

### NGN2 and ASCL1 plasmid construction and production of DNA-loaded PBAE nanoparticles

For efficient conversion of ATGs into multiple subtypes of neuron-lineages, the combination of ASCL1 and NGN2 was selected based on their unique characteristics, including, proneurogenic pioneer activity, promotion of neuronal maturation and synaptic integration, and the ability to redirect regulatory priorities toward the positive regulation of nervous system development and axonal guidance. To produce ASCL1 and Neurog2 plasmids, rat ASCL1 and Neurog2 cDNA were first amplified using a high-fidelity DNA polymerase. The amplified products were subsequently cloned into a pcDNA3.1-based vector following the method as previously described by Xu et al. [48]. For ATG-specific expression, the rat promoter rGFAP was incorporated into the vector to generate pcDNA3.1-rGFAP-EGFP-IRES-ASCL1-r plasmid. The primer sequences used for RT-PCR amplification of ASCL1 and Neurog2 are detailed in Table S1. After verifying the accuracy of constructed vectors through restriction enzyme analysis and DNA sequencing, pcDNA3.1 plasmids encoding ASCL1 and Neurog2 cDNA were transfected into C6 cell lines using Lipofectamine 2000 transfection reagent. The green fluorescence in cells observed 36 to 48 h post-transfection indicated successful plasmid construction and expression. Subsequently, PBAE-based plasmid loading was formulated as previously reported [46]. Briefly, 5 volumes of plasmid DNA (100 µg/ml) were added dropwise to 1 volume of polymer solution at a PBAE-to-plasmid DNA ratio of 60:1 w/w. Both solutions were adjusted to pH 6.0 using a 0.1 N hydrochloric acid. The resulting DNA-polymer nanocomplexes were washed with 5 volumes of ultrapure water and concentrated to 1 mg/ml of plasmid DNA using ultracentrifugal filters, as quantified by the Quant-iT™ PicoGreen^®^ dsDNA Assay Kit. Furthermore, DNA integrity was determined by dissolving the particles in dichloromethane anhydrous (DCM) followed by extraction into PBS. Samples were electrophoresed on a 1.2% agarose gel next to unprocessed plasmid DNA. Densitometry analysis was performed using ImageJ analysis software.

### In vitro transfection efficiency of NGN2/ASCL1-loaded PBAE nanoparticles

To achieve robust reprogramming efficiency, we first optimized the delivery ratio of ASCL1- and NGN2-encoding plasmids. Preliminary optimization experiments comparing 1:1, 2:1, and 1:2 ratios (with constant total DNA amount) revealed that the 1:1 ratio yielded the highest neuronal conversion, approximately ~ 20% increase over other two ratios. Subsequently, different types of DNA-loaded PBAE nanocomplexes, each carrying 1 µg pcDNA3.1 plasmid DNA, were introduced into the astroglial wound model at a concentration of 1 µg/well. Following a 5-hour incubation, the culture medium was replaced with fresh high-glucose DMEM supplemented with 10% fetal calf serum and 1% G5 supplement. After an additional incubation of 48–60 h, the medium was removed, and lysis was performed using 0.5 ml of TRIzol™ Reagent (Promega, Madison, WI) for total RNA isolation. In parallel, another batch of identically treated cells were transferred to Neurobasal Medium supplemented with 1% B27 supplement, 2µM forskolin (FSK), 10 µM SB431542, and 1 mM VPA continuously maintained for 5 d, 7 d, 14 d, and 21 d. Subsequently, these cells weresubjected to three freeze-and-thaw cycles to ensure complete lysis. The luciferase activityin relative light units (RLU) was measured using a standard Luciferase Assay Kit (Promega) with a 20/20n luminometer (Turner Biosystems, Sunnyvale, CA). The RLU values were normalized to the total protein amount determined by a Bicinchoninic Acid (BCA) Protein Assay Kit (Thermo Scientific, Rockford, IL).

Notably, to investigate the molecular mechanisms of ATG-to-neuron reprogramming following transduction with NGN2/ASCL1-loaded PBAE nanoparticles, we applied inhibitors targeting key pathways. MG132 (10 µM), a selective 26 S proteasome inhibitor targeting Dyrk1B, and harmine (15 µM), a specific Dyrk1 family kinase inhibitor, were used individually or in combination. Both compounds, dissolved in DMSO (final concentration ≤ 0.1%), were introduced to the culture medium prior to reprogramming induction and maintained for 5 days, followed by molecular analyses.

### Immunofluorescence

For immunostaining of different treatment experiments, all cultures were fixed with 4% paraformaldehyde for 20 min, followed by incubation with 2% BSA and 5% corresponding secondary antibody host serum in PBS for one hour at room temperature (RT). Primary antibodies diluted in 1% BSA in PBS were incubated with the specimens overnight at 4 °C. The following primary antibodies were used: goat anti-GFAP (1:200), rabbit anti-CSPG (1:200), mouse anti-Tuj-1 (1:200), rabbit anti-Brevican (1:300), rabbit anti-SYN (1:400), and rabbit anti-GFP (1:800). After removal of the primary antibodies and thoroughly washing three times with PBS, the cultures were incubated with the appropriate secondary antibodies conjugated to various fluorescent labels, such as Alexa Fluor 488-conjugated Affinipure anti-mouse IgG and anti-rabbit IgG as well as Alexa Fluor 594-conjugated Affinipure goat anti-mouse IgG and anti-rabbit IgG, for 2 h in the dark at RT. The nuclei were counterstained with DAPI. Finally, the coverslips were mounted onto glass slides utilizing an anti-fade mounting medium. For the investigation of relevant molecular mechanisms, the following antibodies were used: mouse anti-Cend1 (1:200), rabbit anti-Cyclin D1 (1:400), rabbit anti-Dyrk1B (1:100), and RanBPM (1:200). The staining procedures were consistent with those described above. To evaluate the in vivo genetic reprogramming of ATGs into neurons following the delivery of PBAE nanoparticle-loaded *ASCL1* and *NGN2* genes, immunostaining of the spinal cord sections (*n* = 3 per group) was performed at 2, 3, and 4 weeks after injection of PBAE nanoparticles into GS according to a previously established protocol [30]. Notably, animal intraperitoneal anesthesia was performed using the same procedure as described above until non-responsiveness to noxious stimuli, followed by transcardial perfusion with 0.9% saline and then 4% paraformaldehyde. Finally, spinal cord sections were mounted onto glass slides for observation using confocal microscopy. The number of cells expressing various markers was then counted manually using the same method as described previously [49]. Notably, positive cells were reported as an average count per 0.5 mm^2^ across 15 randomly selected fields of view per coverslip observed with a fluorescence microscope.

### Flow cytometry

Cell cycle progression was determined by flow cytometry (Profile II; Coulter, Brea, CA, USA) according to a previously described protocol [50]. Briefly, after ATGs were subjected to above-mentioned PBAE-NGN2/ASCL1 transduction and neural induction treatment, the cells were trypsinized and gently triturated to single-cell suspensions. These suspensions were then filtered through 80-μm nylon mesh prior to analysis. Concomitantly, the cells were fixed in 70% ethanol and treated with 10 μL of RNase A (5 mg/mL) at 37 °C for 30 min, followed by staining with propidium iodide (100 µg/mL) for an additional 30 min. Subsequently, the cells were washed three times with PBS containing 10% FCS and 0.02% sodium azide before flow cytometry analysis. Finally, the proliferation index (PI) was calculated and compared based on cytometry data.

### Reverse transcription PCR and quantitative PCR analysis

Total RNA was extracted from in vitro GS cells undergoing the treatment of PBAE nanoparticle-loaded ASCL1 and NGN2 plasmids using RNAeasy (Qiagen), according to the manufacturer’s instructions. One microgram of total RNA was reverse-transcribed into cDNA using the PrimeScript RT reagent kit (Takara), and ten nanograms of cDNA were used for PCR amplification. Concomitantly, quantitative RT‒PCR was performed in triplicate for each sample and repeated in 3 independent experiments. The genes of interest analyzed included DCX, Tuj-1, NeuN, CHAT, Cend1, Brn3a, Transgelin, GFAP, and CSPG. mRNA levels were quantified via SYBR green-based quantitative real-time PCR (Takara) using an ABI Prism 7900 HT (Applied Biosystems, USA). The housekeeping gene glyceraldehyde 3-phosphate dehydrogenase (GAPDH) served as an internal control for normalizing gene expression relative to both RNA and the target gene expression levels compared to the control. The data are presented as fold change relative to the target gene/GAPDH ratios. All results were validated through at least three independent assays. The qPCR primer sequences are listed in Supplementary Table S1.

### Western blots

Cells subjected to different treatments were lysed in RIPA buffer on ice for 30 min. The protein extracts were sonicated and centrifuged, and the resulting clarified lysates were collected. Protein concentration was determined using a bicinchoninic acid (BCA) assay. Western blotting was subsequently performed as previously described [21]. The following primary antibodies were used: GFAP, Brevican, CSPG, DCX, Tuj-1, BrdU, SYN, Cyclin D1, NeuN, Brn3a, Transgelin, CHAT, EDU/β-actin, CEND1, Dyrk1B, RanBPM, Notch1, β-Actin, and γ-tubulin. All primary antibodies were diluted according to the manufacturer’s instructions or a minor adjustment. β-Actin or γ-tubulin served as an internal control (detailed antibody information is provided in Supplementary Table S2). After thorough washes with PBS, the immunoblots were visualized using enhanced chemiluminescence and imaged with a gel documentation system. Densitometric analysis of bands was performed based on at least 3 independent replicates.

### RNA-seq analysis

The global gene expression profile of the following cell populations was analyzed by RNA-seq: GS cells, pri-neurons, and induced GS cells (7, 14, and 21 days after PBAE nanoparticle-loaded ASCL1 and NGN2 transduction). Total RNA from each group was extracted using an RNeasy mini kit (TaKaRa) according to the manufacturer’s instructions. Ribosomal RNA (rRNA) was depleted prior to RNA-seq library preparation. The prepared libraries were sequenced using an Illumina HiSeq3000 sequencer (Wuhan Kangce Biotechnology Corporation, China). Subsequent steps were undertaken as described previously [51]. The raw sequence reads were trimmed and aligned to the rat reference genome using TopHat. Transcript assembly and differential expression analysis were conducted using Cufflinks. Scatterplots of gene expression levels were generated as described in a previous report [52]. The biosample accession number for the RNA-seq data file reported in this study is GSE319791 and provided at https://www.ncbi.nlm.nih.gov/geo/info/update.html.

### Calcium imaging

To better evaluate the functional properties of ATG-derived neuron-like cells, Calcium influx of individual cells was measured as previously described [48, 53]. In brief, three weeks after treatment with PBAE NP-loaded A/N plasmids and subsequent neural induction, differentiated cells in the GS regions were incubated with 2 µM Fluo-2 AM in Neurobasal medium with 1% B27 supplement for 30 min at 37 °C. The cells were then thoroughly washed twice with extracellular medium (EM, 140 mM NaCl, 2 mM CaCl_2_, 5 mM KCl, 10 mM HEPES, and 10 mM glucose, adjusted to pH 7.2–7.4). Calcium imaging was conducted at 37 °C in EM with a confocal scanning microscope (Zeiss) equipped with a perfusion system. To specifically evaluate Ca^2+^ influx, the addition of either a Ca^2+^ channel activator (10 µM Bay K, Sigma) or blocker (5 µM nifedipine, Abcam) to cultures was conducted to monitor corresponding changes in Ca^2+^ influx. The entire procedure followed our previously reported protocol [53].

### Electrophysiology

Three weeks after transduction of ATGs with PBAE nanoparticle-loaded A/N plasmids and subsequent neural induction, the differentiated cells in the GS region were transferred to artificial cerebrospinal fluid (ACSF) and equilibrated with 95% O_2_ and 5% CO_2_ at 37 °C for 15 min to assess their ability to fire action potentials. The ACSF was made as previously described [54]. Whole-cell patch-clamp recordings were then conducted at 20–22 °C on cells within the GS region that were identified visually and electrophysiologically. In current-clamp mode, current pulses were applied with a step size of 10 pA to evaluate the capacity of the cells to generate action potentials as previously described [55]. Whole-cell sodium and potassium currents were elicited and recorded under voltage-clamp mode in response to a series of voltage steps ranging from − 50 to + 50 mV in 10 mV increments (*n* = 20). Finally, data analysis was carried out using pClamp9 Clampfit software and MiniAnalysis software.

### Animal SCI model and in vivo injection

Thirty adult SD rats (200–250 g) were used as recipients and randomly divided into three groups (*n* = 10 per group): (1) Normal control (no SCI); (2) SCI + delivery of PBAE NP-loaded empty plasmid; (3) SCI + delivery of PBAE NP-loaded A/N plasmids. Following intraperitoneal anesthesia with 1% sodium phenobarbital (50 mg/kg) until non-responsiveness to noxious stimuli, the SCI model was developed using a modified Allen method [56]. One week after SCI, the modeled animals exhibiting complete hindlimb paralysis and loss of function were selected for intralesional injection. Under stereotaxic guidance, either 4 μL of PBAE NP-loaded A/N plasmids or an equal volume of saline alone was injected into the GS core site of the injured spinal cord. Notably, injections were performed using a 10 µL siliconized Hamilton syringe with a pulled, beveled glass pipette tip (80 μm inner diameter) connected to a syringe pump (Pump 11 Elite Nanomite Syringe Pump; Reward apparatus). The injection rate was set at 0.5 µL/min, and the needle was kept in place for an additional 5 min to minimize backflow upon withdrawal. Control animals received the same volume of saline or PBAE NP-loaded empty vector plasmid. After injection, all rats received a daily subcutaneous injection of two antibiotics, penicillin (10^4^ U) and gentamicin (8 × 10^4^ U) for at least 3 days to prevent urinary tract infection.

### Behavioral assessment and evoked potential

To evaluate the therapeutic effect of the delivery of PBAE NP-loaded A/N plasmids in SCI rats, we conducted a series of behavioral assessments following the injection of PBAE NP-A/N into the SC region of the lesioned spinal cord. The behavioral tests, including the BBB Score, Rump-height index (RHI), cylinder test, gait analysis, and evoked potentials test, were conducted. These methods are relatively safe, feasible, and highly sensitive to subtle alterations in spinal cord functions. All behavioral procedures were performed as previously described [57, 58]. Notably, only animals with stable or pronounced neurological deficits were included in the behavioral tests. At least five rats showing behavioral alterations were selected for each group (sham group and group receiving PBAE NP-loaded A/N plasmid). In addition, all tested rats were required to receive a short-term habituation training to adapt the testing environments or equipment prior to formal evaluation.

### BBB Scale

The Basso, Beattie, and Bresnahan (BBB) locomotor scale was employed to assess behavioral outcomes in rats with SCI. The analysis of hindlimb functional recovery was performed at 1, 3, 5 and 7 weeks after the delivery of PBAE NP-A/N plasmids into GS in the lesioned spinal cord as previously described [59, 60]. Notably, functional scores were independently recorded by two observers blinded to the experimental groupings. The observers are required to determine the animal behaviors involving the rat’s ambulation, gait, limb movement coordination, paw position and space, tail activity, and body stability in an open, noise-free field for 5 min after rats gently familiarize themselves to the field. Locomotor functions were scored weekly from day 1 up to 7 weeks after the delivery of PBAE carrying the two TFs. The BBB scale was specifically developed to quantify locomotor recovery in SCI rats, with total scores ranging from 0 to 21 in the light of the complexity of hindlimb motor performance. In this study, the scores were subdivided into three categories: (i) a BBB score less than 7 was used for assessment of movement of individual joints in rat hindlimbs, (ii) a score between 8 and 13 was used to reflect improvements in limb gait and coordination, and (iii) a score above 14 indicated the recovery of fine motor skills.

### Rump-height index assay

The Rump-height index (RHI) is applied to assess the ability of animals to support their body weight following different treatments. Movements of SCI rats were observed and recorded as they walked across a runway beam from the left to right side at the corresponding time points consistent with those used for BBB scoring, after delivery of PBAE NPs carrying the A/N. RHI is defined as the height of the rump normalized to the thickness of the beam, measured along the same vertical axis. To minimize variability in pre-surgery RHI values of each rat, RHI values were standardized as previously reported [61].

### Cylinder test

To better evaluate functional recovery in SCI rats following injection of PBAE NPs-A/N into GS region of the lesioned spinal cord, animals were placed in an open-top transparent plastic cylinder (50 cm in diameter, 50 cm in height). Their forelimb activity during rearing against the cylinder wall was recorded according to previously described methods [58]. In the cylinder test, the forelimb used is defined by the placement of the whole forepalm against the wall of the arena. Each rat was recorded 5 times daily for 3 min each session, over three consecutive days. Notably, the testing environment must be quiet to avoid distractions and potentially freezing behavior in the animals caused by loud noises and conversation. Functional locomotor assessment was performed in at least 5 SCI rats over a two-week period after injection.

### Gait analysis

To further identify the improvements in motor function and coordination in the SCI rats following injection of PBAE NP-A/N, the forelimbs and hindlimbs of the tested rats were painted, and each animal was individually placed into a glass walkway (10 cm wide, 80 cm long). The animals were allowed to move freely in both directions and were required to complete three compliant runs for gait analysis. The Noldus Catwalk XT (version 10.5), an automatic quantitative gait analysis system, was used to detect several subtle dynamic changes of a rat’s walk and identify neurological gait abnormalities. Footprints were photographed as each rat voluntarily traversed a glass plate toward a small dark shelter located at the end of the platform. Gait-related parameters such as stride pattern, individual walking speed, and distance duration were recorded and analyzed using the Catwalk XT 10.5 system in accordance with the manufacturer’s guidelines. In addition, pressure applied by each paw was calculated based on footprint intensity and partially on the area covered by the footprint. Of note, a total of 10 SCI rats underwent functional gait assessment after

injection of PBAE-A/N NP.

### Ledged beam test

To evaluate the improvement of sensorimotor deficits in an SCI rat model following the delivery of PBAE NPs carrying the two TFs, the tested animals were placed on a tapered ledge beam with a width gradually decreasing from 6 cm at the start to 1 cm at the end. Their movement along the beam to the desired direction was observed and recorded. The experimental procedures and the data analysis were performed in accordance with previously described methods [62]. Notably, all tested rats must undergo a short-term habituation training to familiarize themselves with the test environment, followed by 5 pretrials on the grid-surfaced beam prior to formal assessment.

### Motor and somatosensory evoked potentials

To further validate functional recovery following the delivery of PBAE NPs carrying the two TFs, both sensory and motor tracts of the injured spinal cord were measured 7 weeks after injection of PBAE NPs into the GS region (at least 10 rats in each group), as previously described [63]. Briefly, 1, 3, 5, and 7 weeks post-surgery and subsequent respective treatments, rats were randomly assigned to SEP or MEP recordings, with evaluators blinded to treatment conditions. Animals were anesthetized via subcutaneous injection of pentobarbital sodium (30 mg/kg). Notably, the stimulation point was located at the middle of the tibialis anterior muscle group. Subsequently, a 0.8-mm stimulating electrode was positioned at 1 cm distal to the tibial muscle, and a continuous square-wave stimulus was undertaken 20–30 times. Stimulation parameters were set according to our previous report [64]. For MEP recordings, the same cohort of rats was subsequently used for SEP measurements following MEP testing. At this point, a stimulating electrode was inserted into the contralateral gray matter of the spinal cord adjacent to the motor tract, while a recording electrode was inserted into the central tibialis anterior muscle and a reference electrode was positioned 1 cm ipsilaterally to the recording electrode. Single-stimulus intensity was 1 ~ 5 mV. Of note, during MEP and SEP recordings, rat tails were immersed in saline to ensure optimal signal conduction. Both MEPs and SEPs are characterized by latency unit (ms) amplitude (mV). The evoked potential meter (4-channel Nicolet EDX EMG) and evoked potential recorder (Neuropac-II) are from the United States.

### Statistical analyses

All data are presented as mean ± SEM from at least three independent experiments, unless otherwise stated. Statistical analyses were conducted using SPSS 10.0. For two group comparisons, Student’s t-test was used for continuous and independent data, while Kolmogorov-Smirnov test was applied when sample sizes are sufficiently large and variances are equal. For comparisons among multiple groups under different conditions, Tukey’s HSD test for all pairwise comparisons following one-way ANOVA was employed. two-way ANOVA was used for all longitudinal behavioral data. A P-value of less than 0.05 was considered statistically significant.

## Results

### Morphological and biochemical characterization of ATGs and an in vitro glial scar model

**Fig. 1:**
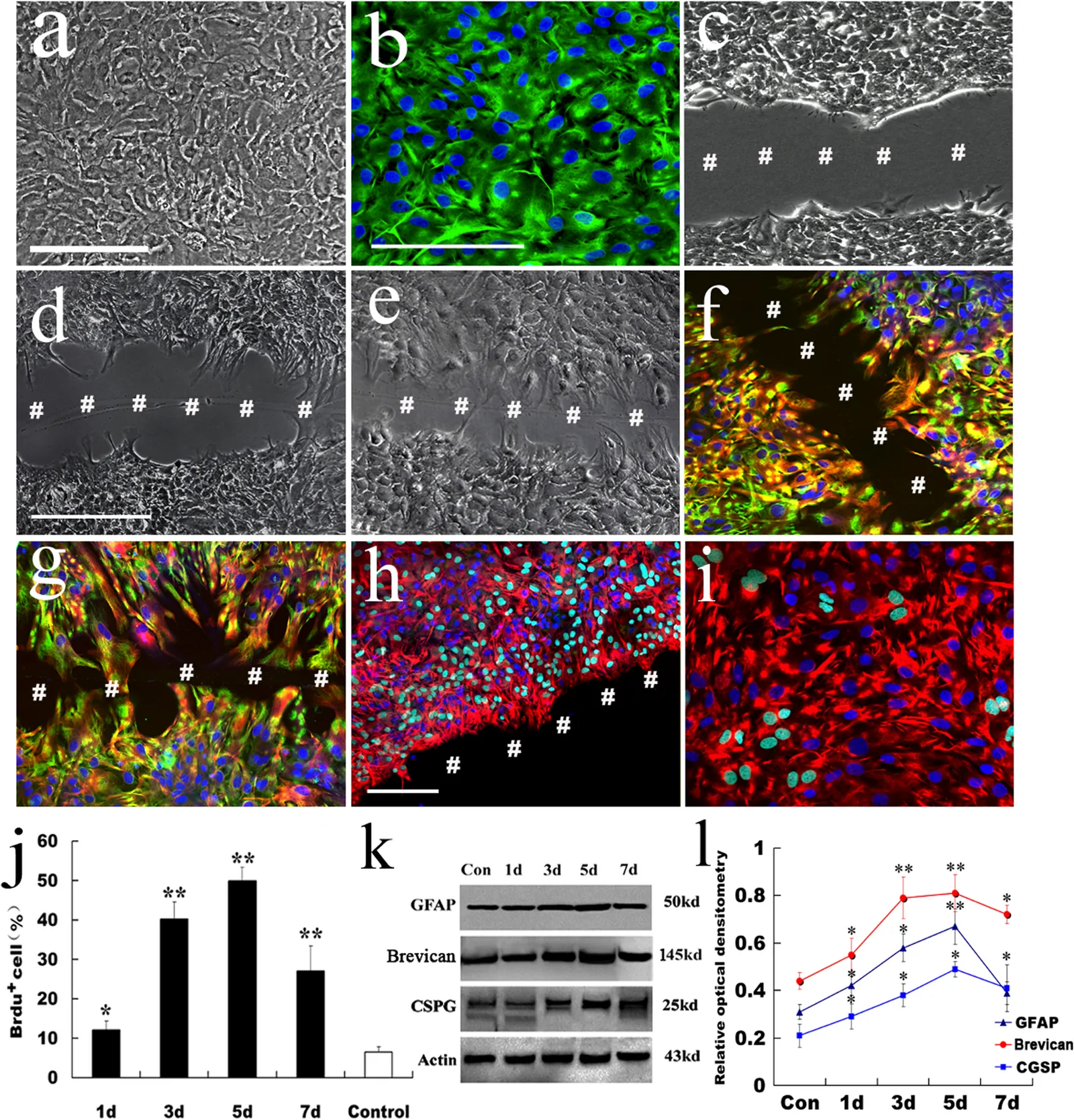
Morphological characteristics of ATGs, identification, and analysis of an in vitro glial scar model. **a** Primary culture of ATGs. **b** Representative photomicrographs of GFAP-immunostained ATGs. **c**–**e** Morphological changes in ATGs at 1, 3, and 5 days after mechanical injury during the establishment glial scar model. **f** Dual immunofluorescence staining of the glial scar for CSPG (red) and GFAP (green) post-modeling. GFAP^+^ cells within the glial scar region coexpressed CSPG, confirming successful GS formation in vitro. **g** Dual immunofluorescence staining of the glial scar for GFAP (green) and brevican (red) after modeling. GFAP-positive cells within the glial scar region coexpressed brevican, further validating GS establishment in vitro. **h** Proliferation of ATGs within the glial scar region (BrdU, green; GFAP, red). **i** Proliferation of normally cultured ATGs (BrdU, green; GFAP, red). **j** Quantification of BrdU^+^ cells in the glial scar region at 1, 3, 5, and 7 days after mechanical injury. The data are reported as the means ± SEM (*n* = 3 independent experiments). *p < 0.05 and **p < 0.01 compared with the corresponding controls. **k** Western blot analysis of the expression of glial scar antigens (CSPG and brevican) and an ATG marker (GFAP) in the glial scar region at the indicated time points. β-actin served as a loading control. **l** Quantification of relative protein expression levels from the immunoblots. The data are presented as the means ± SEM(*n* = 3 independent experiments). *P < 0.05 and **p < 0.01 compared with the corresponding controls. “#” represents the mechanical scratch injury area. Scale bars = 100 μm

To evaluate the efficacy of PBAE-loaded ASCL1 and NGN2 plasmids in converting resident GS cells into neurons, we first cultured, characterized, and identified primary ATGs derived from rat spinal cords. Concomitantly, we developed and characterized an in vitro GS model. As shown in Fig. 1, phase-contrast microscopy revealed that most ATGs exhibited a flat, polygonal morphology and formed a confluent monolayer five days after purification (Fig. 1a). Immunostaining further confirmed that these cells expressed the astrocyte-specific marker glial fibrillary acidic protein (GFAP) (Fig. 1b). As for the in vitro SC model, a monolayer of ATGs was subjected to mechanical scratch-wound injury and exhibited a gap-like denuded area with a relatively neat scratch margin after 24 h (Fig. 1c). At 3 days after scratching, ATGs at the scratch margin showed marked hypertrophy, significant proliferation, and the extension of cytoplasmic processes into the denuded area. Over time, the gap progressively closed, accompanied by cell migration and process elongation (Fig. 1d and e). Furthermore, immunofluorescence staining revealed that cells within the scratch area expressed CSPG and Brevican, two specific markers of GS cells (Fig. 1f and g). In addition to expression of the characteristic GS markers CSPG and Brevican, bromodeoxyuridine (BrdU) incorporation assays revealed a significantly greater number of BrdU-positive cells following mechanical scratch-wound injury (Fig. 1h), whereas only a few BrdU-positive cells were observed in uninjured ATGs (Fig. 1i). The quantitative analysis confirmed a significant increase in the percentage of BrdU-positive cells compared with that in the control group (Fig. 1j). Consistently, Western blot analysis showed the significant upregulation of GFAP, CSPG, and Brevican expression in ATGs within the lesioned area. The expression of these molecules increased in a time-dependent manner albeit with a slight decrease by 7 days after injury (Fig. 1k and l).

### Identification of ASCL1/NGN2 plasmids and characterization of PBAE-DNA plasmid nanoparticles

**Fig. 2:**
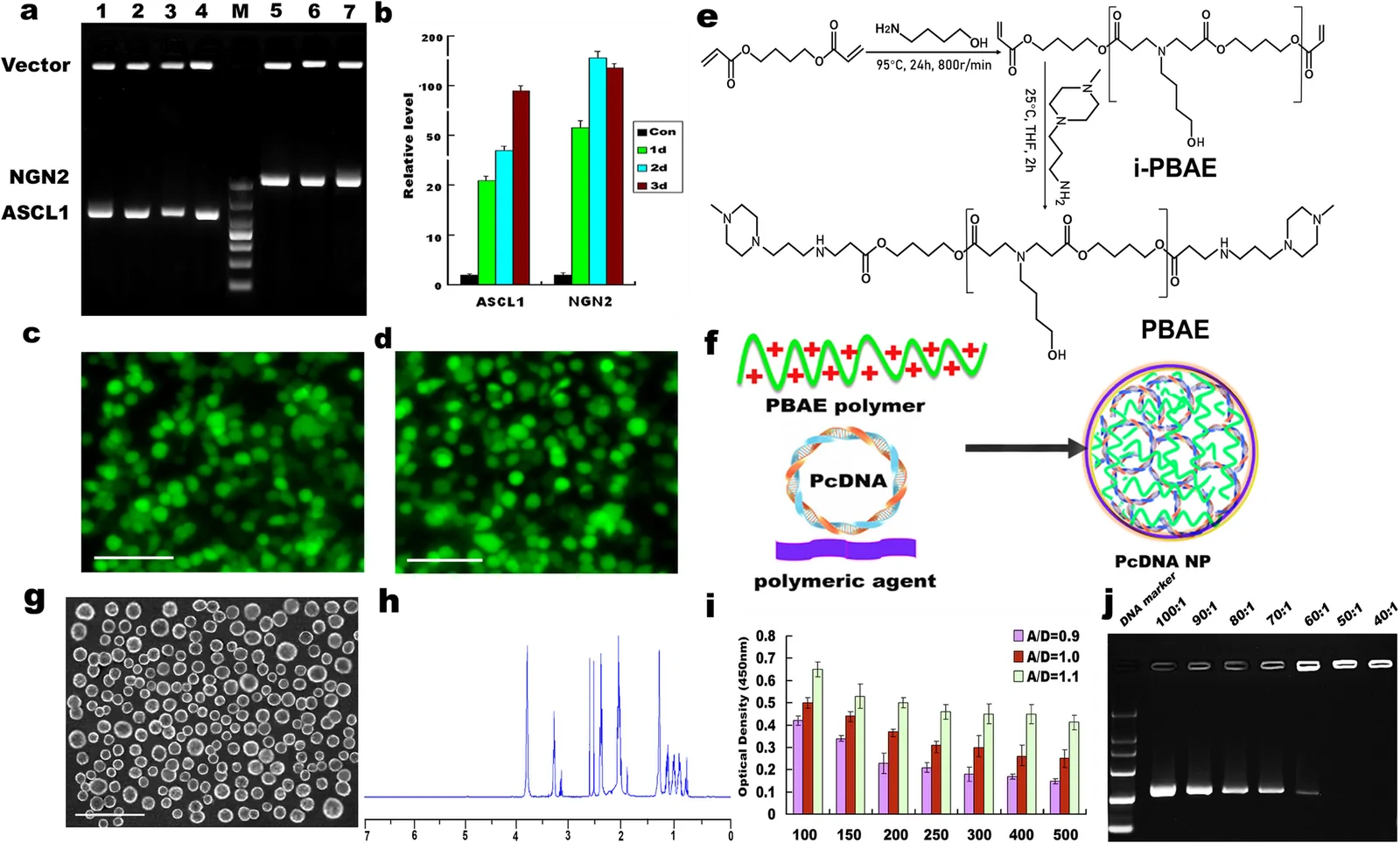
Development of the ASCL1/NG2 plasmid and synthesis and characterization of PBAE-A/N nanoparticles. **a** Identification of recombinant ASCL1/NGN2-pcDNA3 plasmids. Restriction endonuclease digestion confirmed the presence of the *ASCL1* gene in four randomly selected positive clones and the *NGN2* gene in three randomly selected positive clones within the pcDNA3 expression vector. **b** qPCR detection of *ASCL1* and *NGN2* expression in C6 cells at 1 day, 2 days, and 3 days after transfection with the ASCL1/NGN2-pcDNA3 plasmids. **c**, **d** Expression of ASCL1 and NGN2 in C6 cells at 48 h after transfection with the ASCL1/NGN2-pcDNA3.1 plasmid. *Note: No photomicrographs are shown for the control group (transfected with an empty vector), since both expression of both genes was undetectable in C6 cells. Scale bar = 100 μm. **e** Schematic diagram illustrating the synthesis of PBAE-based polymers by a two-step Michael addition reaction using 1,4-butanediol diacrylate and 4-amino-1-butanol. **f** Schematic depicting PBAE/pcDNA complex formation via electrostatic interactions between the PBAE carrier material and the pcDNA payload. **g** Representative ultrastructural visualization of PBAE polymers complexed with ASCL1 and NGN2 expression plasmids, namely, the PBAE/DNA nanoparticles. **h** Analysis of physicochemical characteristics of PBAE nanoparticles complexed with ASCL1/NGN2-pcDNA3 using ¹H NMR spectroscopy and FTIR spectroscopy. **i** Evaluation of the cytotoxicity of PBAE nanoparticles based on the molar ratio (A/D) of the base reaction precursors used in PBAE synthesis. **j** Agarose gel retardation analysis of PBAE/pcDNA complex formation efficiency at different weight ratios of DNA to polymer

We further explored our hypothesis by constructing and sequencing ASCL1 and NGN2 expression plasmids, which were subsequently used to transfect 293T cells. As shown in Fig. 2a, the accurate construction of the ASCL1 and NGN2 expression plasmids was verified by restriction endonuclease digestion and agarose gel electrophoresis. Furthermore, a specific PCR analysis revealed a progressive increase in ASCL1 and NGN2 expression in C6 cells at 1 and 3 days post-transfection (Fig. 2b). Consistent expression patterns for ASCL1 and NGN2 in C6 cells were also observed (Fig. 2c and d). Concurrently, PBAEs were synthesized for plasmid by using a two-step Michael addition reaction involving 1,4-butanediol diacrylate and 4-amino-1-butanol (Fig. 2e). The carrier material PBAE and pcDNA formed complexes through electrostatic interactions, as depicted in Fig. 2f. Furthermore, electron micrographs showed that the PBAE/pcDNA nanoparticle complexes exhibited a roughly spherical morphology, with diameters ranging from approximately 180 to 250 nm (Fig. 2g). The ^1^H NMR spectra and Fourier transform infrared (FTIR) spectroscopy of the synthesized PBAE are shown in Fig. 2h. The hydrogen signal peaks corresponding to the relevant unit of the PBAE structure indicate the successful synthesis of the polymer. Furthermore, the assessment of the toxicity of PBAEs synthesized using three distinct ratios of 4-amino-1-butanol to 1,4-butanediol diacrylate (A/D) indicated that the A/D ratio of 1.1 exhibited the lowest toxicity compared with the other two ratios (Fig. 2i). Finally, nanoparticle complexes of PBAE and pcDNA3.1 at different weight ratios were also identified by a gel retardation assay (Fig. 2j).

### Generation of neurons following the delivery of PBAE-A/N plasmid nanoparticles and conditioned induction

**Fig. 3:**
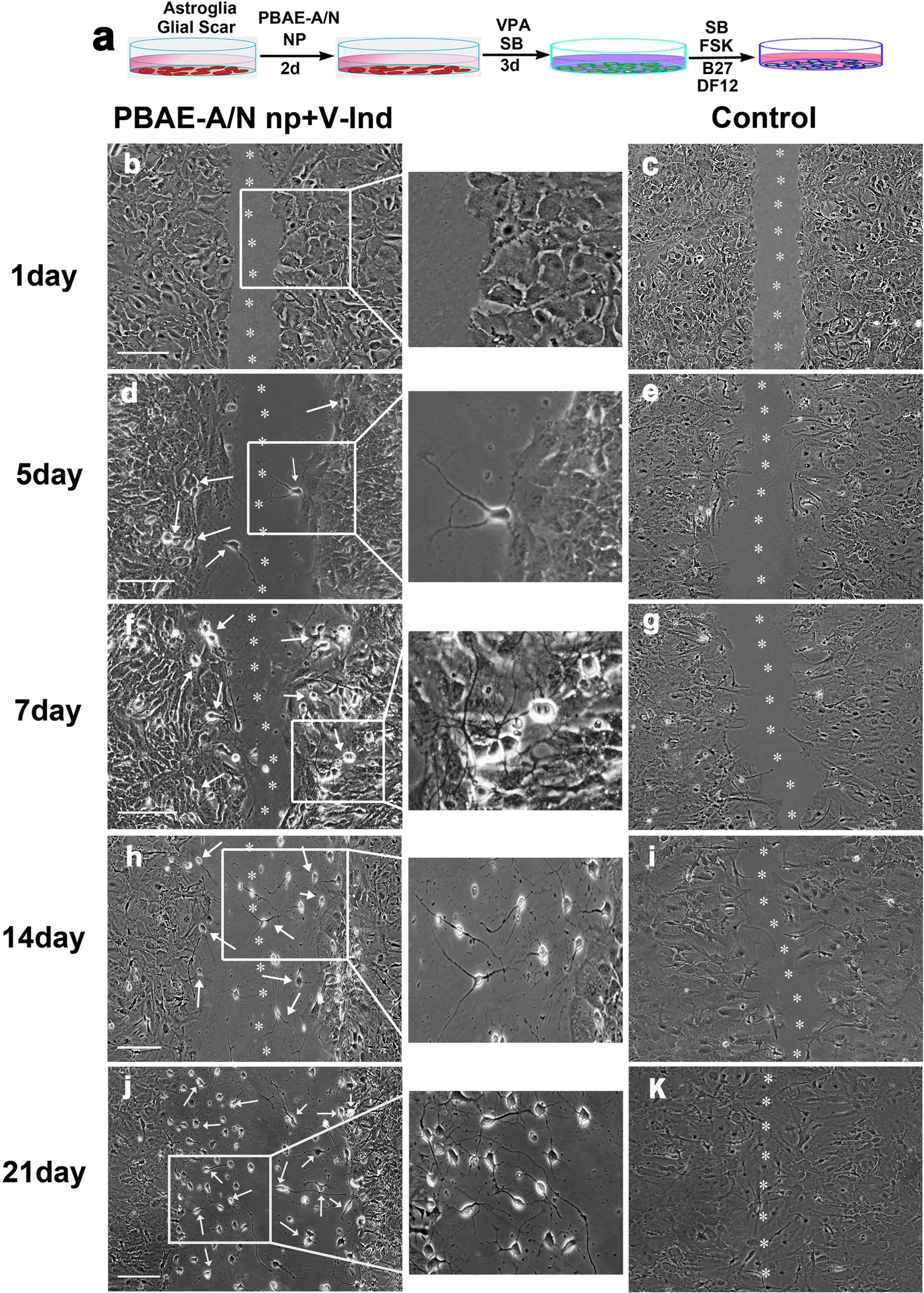
Morphological changes of and reprogramming of PBAE-A/N-transduced ATGs into induced neurons within the glial scar region. **a** Schematic illustration of the experimental strategy for reprogramming ATGs into iNeurons. **b**, **d**, **f**, **h**, **j** Representative phase-contrast images of PBAE-A/N-transduced ATGs within the glial scar region cultured sequentially in standard neuronal medium by preinduction medium for 1, 5, 7, 14, and 21 days. **c**, **e**, **g**, **i**, **k** Phase-contrast images of ATGs transduced with the PBAE-empty vector within the glial scar region and cultured under the corresponding induction conditions. The middle column insets show higher-magnification views of converted cells within the boxed areas. The arrows (↑) highlight representative cells with a neuron-like morphology. Scale bars = 100 μm

To visualize ATG reprogramming into neuronal cells, we administered PBAE-A/N NPs to rat ATG cultures and monitored their morphological changes. A detailed schematic of the manipulation procedure is depicted in Fig. 3a. Subsequently, we tracked the morphology of the reprogrammed cells at various time points following PBAE-A/N NP delivery. As shown in Fig. 3b, no significant morphological changes were observed at one day after delivery; ATGs within the GS area retained their flat, polygonal morphology. By day 5 after delivery, however, an increasing number of neuron-like cells emerged. These cells displayed diverse neurite lengths, closely resembling those of axons or dendrites, and gradually migrated outward from the GS margins as the culture duration increased (Fig. 3d and f). At more than 14 days after delivery, an increased number of neuron-like cells appeared in the lesion margins, with some migrating into the denuded area (Fig. 3h and j). In contrast, the control ATGs infected with PBAE-loaded mock plasmid NPs showed minimal morphological changes and failed to generate neuron-like cells throughout the observation process (Fig. 3c, e and g, and 3i). Remarkably, by day 21 after treatment, many neuron-like cells emerged in both the GS and denuded areas and exhibited substantial morphological alterations (Fig. 3j, with higher magnification views shown in the middle panels). Concurrent observations of control ATG cultures after treatment with PBAE-mock vector NPs between 14 and 21 days revealed no morphological transformation; however, these cultures showed progressive proliferation and migration of ATGs into the denuded area (Fig. 3i and k). These findings suggest that PBAE-A/N NPs effectively enhance the reprogramming of ATGs into neurons.

### Identification of neuron-like cells derived from astrocytes

**Fig. 4:**
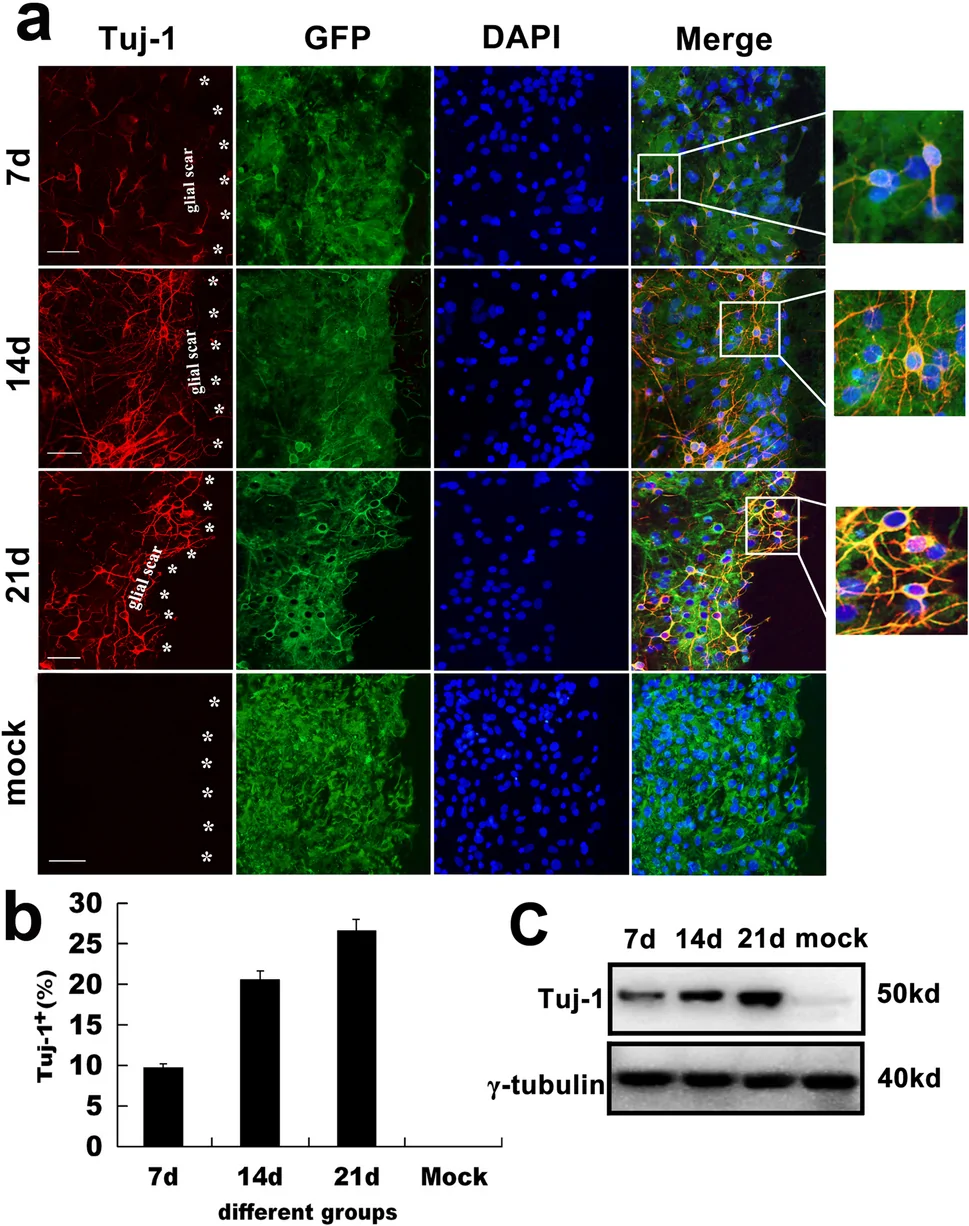
Morphological and biochemical features of ATGs transduced without or with PBAE-A/N within the glial scar region. **a** Representative photomicrographs of immunofluorescence staining showing the expression of Tuj-1 in PBAE-A/N-transduced ATGs at 7, 14, and 21 days, respectively. Asterisks indicate the edge of the glial scar regions. The insets show higher-magnification views of Tuj-1^+^ cells within the boxed areas. Scale bars = 100 μm. **b** Quantification of Tuj-1^+^ cells within the glial scar region. The data are reported as the means ± SEM (*n* = 3 independent experiments). **c** Western blots showing the expression of Tuj-1 in ATGs treated with or without PBAE-A/N for 1–3 weeks. β-actin served as the loading control for total proteins

We evaluated whether the ATG-derived cells acquired neuronal characteristics by performing Tuj-1 immunostaining to confirm their biochemical phenotypes during reprogramming and compared them with those in control groups. Our results revealed a progressive increase in Tuj-1 expression in reprogrammed cells over time. Notably, the number of Tuj-1-positive cells derived from ATGs transfected with PBAE-A/N NPs significantly increased with culture duration. These Tuj-1-positive cells exhibited extended processes and more pronounced arborization by days 14 and 21 after transduction with PBAE-A/N NPs (Fig. 4a, high-magnification images are shown in the right panels). In contrast, no Tuj-1 immunoreactivity was detected in the mock-transfected control group (Fig. 4a, lower panel). Consistent with the immunostaining results, the quantitative analysis revealed that the percentage of Tuj-1-positive cells gradually increased over time following PBAE-A/N NP transfection, showing a 1.0- to 2.5-fold increase across the three specified durations (Fig. 4b). Furthermore, Western blot analysis revealed a time-dependent increase in Tuj-1 expression in reprogrammed cells over the indicated time intervals, whereas Tuj-1 expression did not increase significantly in the cells transfected with the PBAE-mock control (Fig. 4c).

### Effects of the PBAE-A/N plasmids and VPA induction on cell proliferation in the glial scar region

**Fig. 5:**
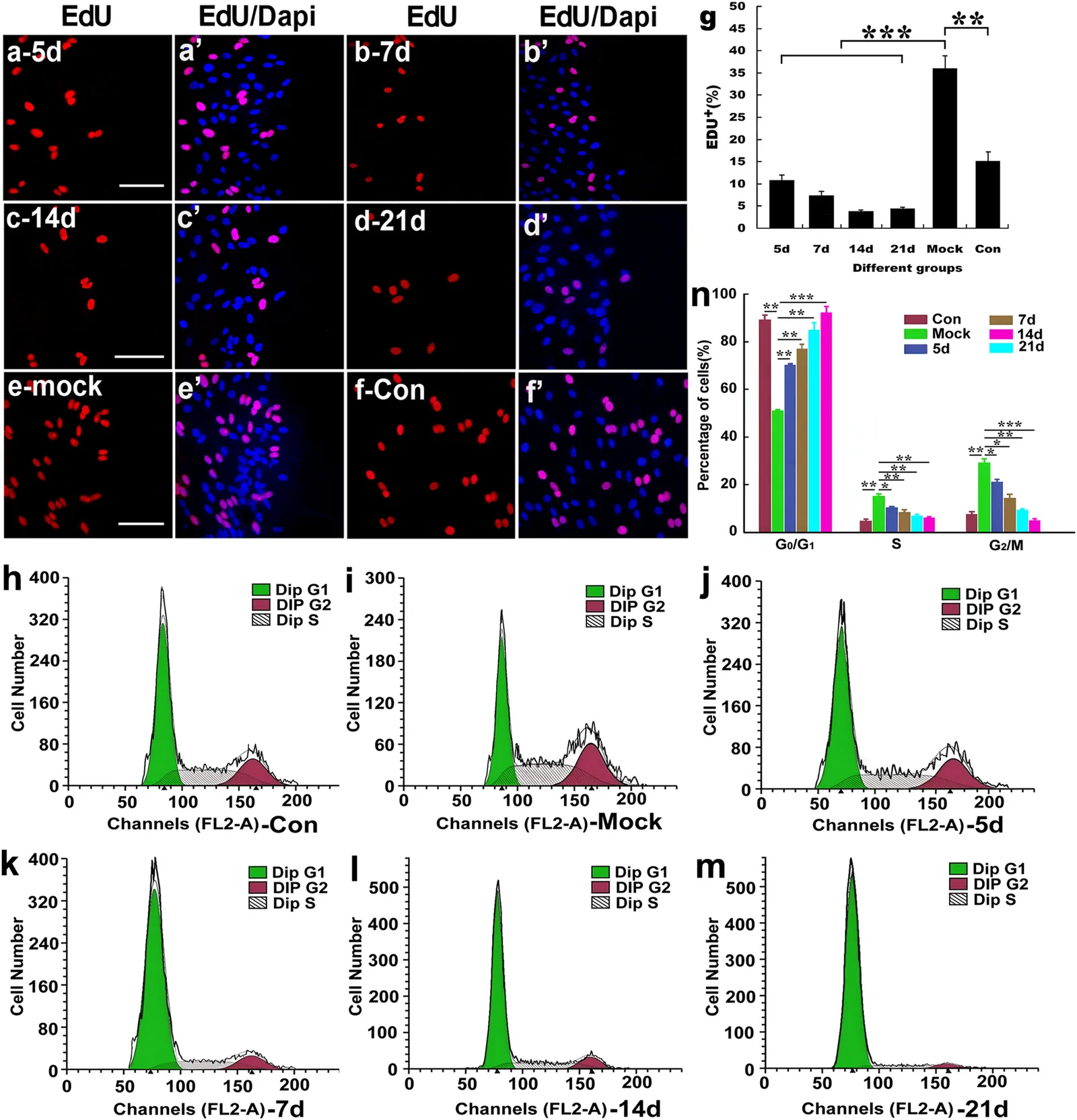
In vitro proliferative capacity and cell cycle analysis of PBAE-A/N-transduced ATGs within the glial scar region. **a**–**f** Representative photomicrographs of EdU incorporation into ATGs after transduction with PBAE-A/N were analyzed to assess proliferation at 5, 7, 14 and 21 days. Compared with those in the mock and control groups, significantly fewer EdU^+^ cells were observed in A/N-transduced samples at the indicated time points. Normal cultured ATGs served as the control; mock represents ATGs transduced with the PBAE-empty vector. Scale bars, 150 μm. **g** Quantification of EdU-positive cells among cells after the indicated treatments. The data are reported as the means ± SEM (*n* = 3 independent experiments). **p < 0.01 and ***p < 0.001 compared with the corresponding controls. Notably, EdU incorporation was significantly reduced in PBAE-A/N-transduced samples within the glial scar region in a time-dependent manner. **h** Cell cycle distribution of normal ATGs. Phases: G0/G1, S, and G2/M. The data are shown in the panel. **i** Cell cycle distribution of ATGs transduced with the PBAE-empty vector. Phases: G0/G1, S, and G2/M. The data are shown in the panel. **j**–**m** Cell cycle distribution of PBAE-A/N-transduced ATGs at 5, 7, 14, and 21 days, respectively. **n** Statistical analysis of the percentages of cells in each phase (G₀/G₁, S, and G₂/M) revealed a significant difference between the PBAE-A/N-transduction group and the control/mock groups. The percentages were calculated using ModiFIT software. The data are presented as the means ± SEM (*n* = 3 independent experiments). *p < 0.05, **p < 0.01, and ***p < 0.001 compared with the relevant controls

In addition to the acquisition of neuronal morphology and biochemical phenotypic characteristics, a significant reduction in cell proliferation is a critical hallmark of ATG-to-neuron reprogramming. Therefore, cell proliferation was assessed and quantified using 5-ethynyl-2’-deoxyuridine (EdU) incorporation assays. Compared with that in the control group, the number of EdU-positive cells was significantly decreased between 5 and 21 days after transfection with PBAE-A/N NPs, (Figs. 5a-f). The quantitative analysis revealed a significant difference in the percentage of EdU-positive cells among the three groups. Transfection with PBAE-A/N nanoparticles resulted in percentages of 10.81% ± 0.53% on day 5, 7.29% ± 0.49% on day 7, 3.9% ± 0.18% at 14 days, and 4.19% ± 0.23% on day 21. In contrast, transfection with the PBAE-mock vector resulted in a percentage of 36.21% ± 3.7%, while the percentage in the untreated control group was 15.7% ± 3.61% (Fig. 5g) (**p < 0.01 and ***p < 0.001 compared with the corresponding controls). The percentage of proliferating cells progressively decreased among the four specified time points, suggesting that PBAE-A/N facilitates the neuronal reprogramming of ATGs. To further substantiate this assertion, flow cytometry was employed to evaluate cell proliferation by monitoring cell-cycle progression. The results revealed distinct alterations in the ATG cell cycle at 5, 7, 14, and 21 days post-treatment (Figs. 5h–m). Consistent with the observations from EdU staining, transduction with PBAE-A/N NPs led to substantial reduction in the proportions of reprogrammed ATGs in both S and G2/M phases, accompanied by a concomitant increase in the G1-phase population at each time point examined. This redistribution of cell cycle phases differed significantly between reprogrammed and control ATGs. Notably, the percentage of PBAE-A/N-transfected ATGs in the G2/M phase was significantly lower than that in both the untreated control and PBAE-empty vector (mock) groups (**p < 0.01, ***p < 0.001; Fig. 5n). Collectively, these data show that PBAE-A/N transduction effectively induces cell cycle exit through G1/S phase arrest, indicating terminal cell cycle arrest and a diminished self-renewal capacity.

### Phenotypic characteristics of converted astrocytes within the glial scar region

**Fig. 6:**
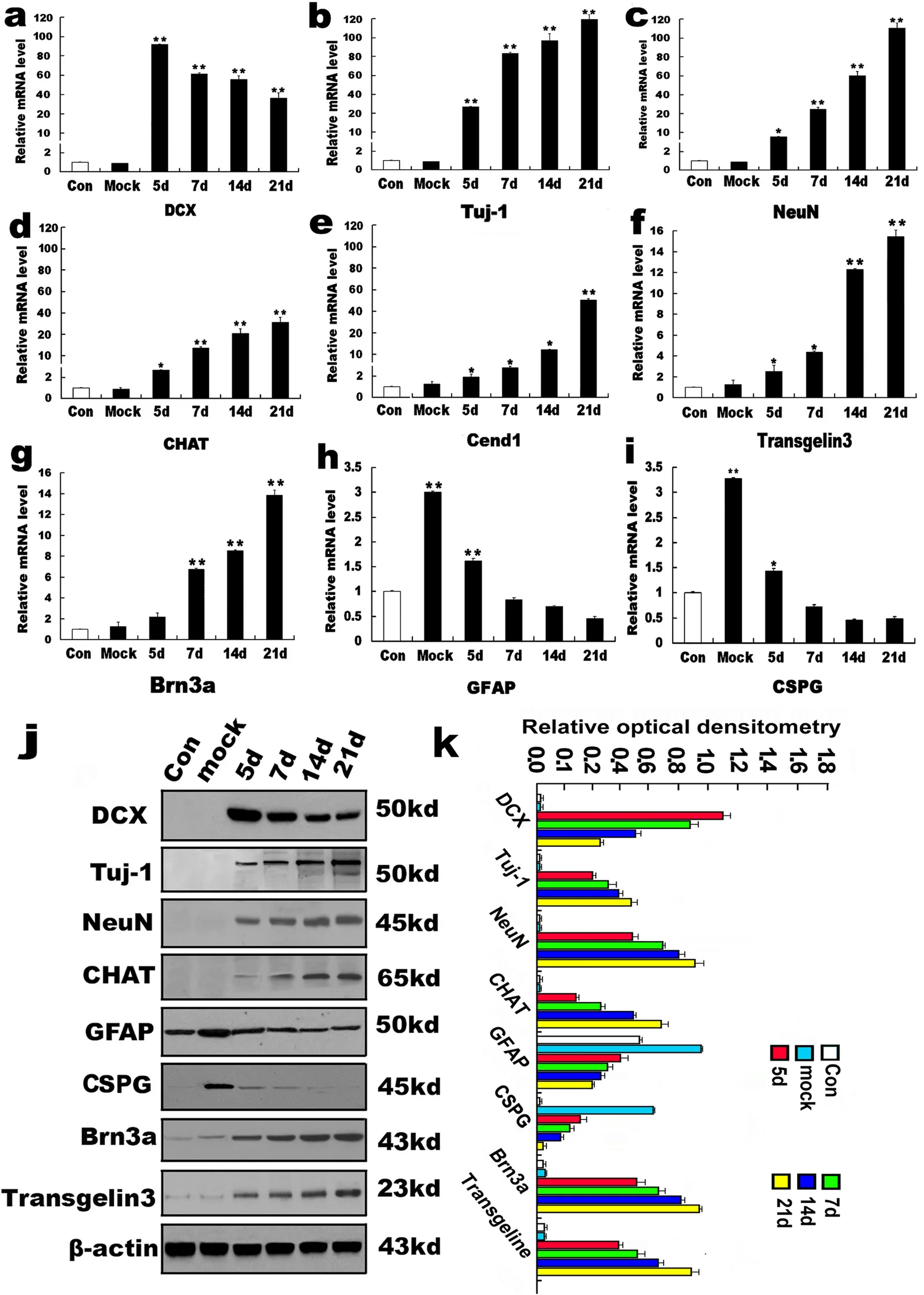
Phenotypic characteristics of ATGs after PBAE-A/N transduction at the transcriptional and translational levels. **a**-**i** RT‒PCR analysis of *DCX*,* Tuj-1*,* NeuN*,* CHAT*,* Cend1*,* Transgelin3*,* Brn3a*,* Gfap*, and *CSPG* transcripts levels for in ATGs that were either nontransduced or transduced with PBAE-A/N for 5, 7, 14, or 21 days. Gapdh served as the loading control for total RNA. The data are reported as the means ± SEM (*n* = 5 independent experiments, *p < 0.05 and, **p < 0.01 by one-way ANOVA with post-hoc test). **j** Western blot analysis showing the protein expression levels of the aforementioned markers (DCX, Tuj-1, NeuN, CHAT, Cend1, Transgelin3, Brn3a, Gfap, and CSPG) in PBAE-A/N transduced ATGs at the indicated time points. **k** Quantification of the expression of the DCX, Tuj-1, NeuN, CHAT, Cend1, Transgelin3, Brn3a, Gfap, and CSPG protein normalized to that of β-actin. The data are reported as the means ± SEM (*n* = 3 independent experiments)

To rigorously address whether this specialized gene delivery system is sufficient for the reprogramming of astroglia into neuronal cells, we conducted an in-depth analysis of a subset of key molecular markers associated with the transition to various neuronal subtypes and relevant regulatory factors at both the mRNA and protein levels using quantitative PCR and Western blotting at the indicated time points. As shown in Fig. 6, following induction with PBAE-A/N nanoparticles, the expression of the neuronal progenitor marker DCX showed significant initial upregulation, albeit with a gradual decline from days 5 to 21 (Fig. 6a). Concurrently, the expression levels of neuronal markers Tuj-1, NeuN, and CHAT progressively increased over a three-week period (Fig. 6b–d). Additionally, the expression of the regulatory markers Cend1, Transgelin3, and Brn3a increased at the indicated time points (Fig. 6e–g). More importantly, the ATG-specific marker GFAP and the glial scar marker CSPG were substantially downregulated in a time-dependent manner and were somewhat more pronounced than that in both the normal and PBAE-mock vector-transduced controls (*p < 0.05, **p < 0.01, and ***p < 0.001, Fig. 6h and i). In line with the qPCR results, Western blots further showed similar trends in the levels of the aforementioned markers from days 5 to 21 after PBAE-A/N transduction (Fig. 6j). Specifically, the introduction of PBAE-A/N NPs to the GS culture significantly increased the levels of neuronal markers. In contrast, notable reductions in GFAP and CSPG expression were observed (Fig. 6j). The quantitative analysis revealed significant changes in the levels of these proteins among the groups (Fig. 6k).

### Transcriptomic profiles of A/N-transduced ATGs

**Fig. 7:**
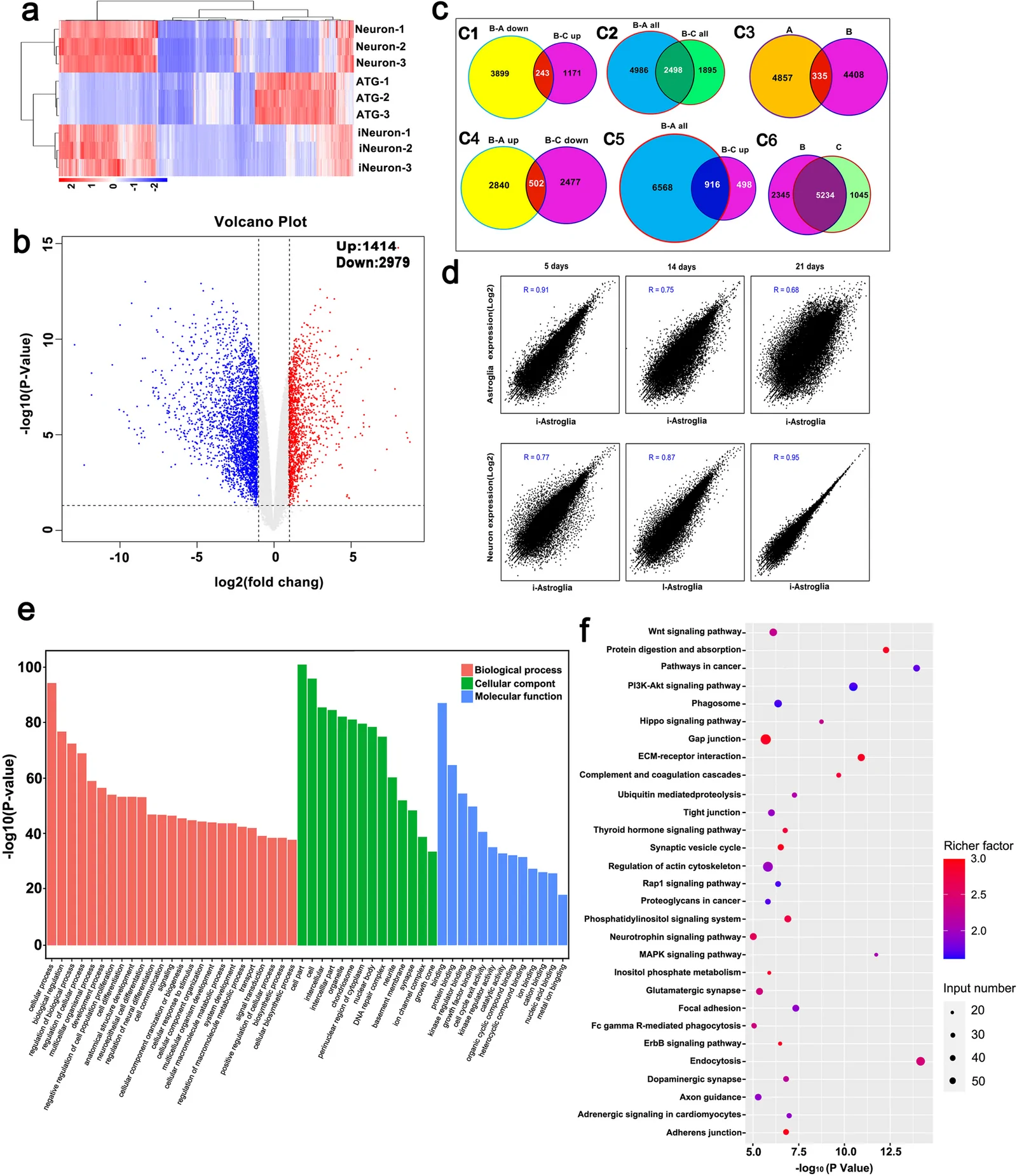
Global gene expression profiles of ATGs, iNeurons (PBAE-A/N-transduced ATGs), and control neurons. **a** Heatmap representing the global expression profile of astrocytes, iNeurons (PBAE-A/N-transduced ATGs at 14 days), and primary neurons classified into the same hierarchical cluster. **b** Volcano plot of showing significantly differentially expressed genes (DEGs, fold change ≥ 2) between PBAE-A/N-transduced ATGs and neurons. The blue and red dots indicate significant DEGs. **c** Venn diagram of differentially expressed genes shared between ATGs, iNeurons (PBAE-A/N-transduced ATGs), and neurons. The differential genes are indicated. Note: A, ATGs; B, PBAE-A/N-transduced ATGs; C, neurons. B-A, B vs. A; B-C, B vs. C. **d** Pairwise scatter plot showing the global gene expression profiles of induced astroglia (PBAE-A/N-transduced ATGs) and neurons. The transcriptome of each cell type was profiled by RNA-seq. Gene expression levels (FPKM) are depicted on a log 10 scale. Pearson’s correlation coefficients (r) are indicated. **e** Enriched biological process, cellular component, and molecular function GO terms for genes overlapping between iNeurons (PBAE-A/N-transduced ATGs) and ATGs. **f** KEGG pathway enrichment analysis. Each circle represents a pathway, color indicates the p-value and the size of the circle indicated the number of genes involved

To further characterize the identity of the iNeurons from PBAE-A/N NP-transduced ATGs within the GS, we profiled the transcriptomes of ATGs, iNeurons, and primary neurons using RNA-seq. Hierarchical clustering analysis revealed a high degree of similarity between iNeurons and neurons but significant differences between these populations and ATGs (Fig. 7a). The volcano plot showed that PBAE-A/N transduction induced substantial changes in gene expression in ATGs compared with neurons, with 1,414 genes upregulated and 2,979 genes downregulated (*p* < 0.05; Fig. 7b). As a method to investigate the reprogramming status of ATG, Venn diagrams were constructed to depict the distributions of upregulated and downregulated genes in ATGs, iNeurons from A/N-transduced ATGs, and neurons (Fig. 7c). A total of 7484 genes (3342 upregulated and 4142 downregulated) were significantly different between ATG and iNeurons; 754 genes (243 upregulated and 502 downregulated) were shared between the populations. In contrast, up to 4383 genes (1414 upregulated and 2978 downregulated) were significantly differentially expressed between iNeurons and neurons, and 2498 shared genes were observed between all differentially expressed genes in iNeurons compared with those in ATGs and those in neurons under standard conditions. Notably, an analysis of the global expression profiles of ATGs and iNeurons (PBAE-A/N transduction at 21 days) revealed more genes with the same expression pattern (5234) between iNeurons and neurons, whereas relatively fewer genes (335) were shared between ATGs and iNeurons (Fig. 7c). In agreement with these results, the pairwise scatter plot shows gene expression levels detected in ATGs, iNeurons, and neurons; iNeurons and neurons were, albeit somewhat distinct, more similar to each other but were highly divergent from ATGs (Fig. 7d). Notably, the similarity between neurons and PBAE-A/N transduced ATGs was gradually increased over time (5, 14, and 21 days). The GO analysis of the co-DEGs to elucidate the functions affected by PBAE-A/N transduction revealed that the overlapping genes in the biological processes were significantly enriched for neural development-related events such as cell proliferation, neurogenesis, regulation of neuronal differentiation, DNA repair, axonogenesis, synapse formation, and growth cone generation. Additionally, enrichment was observed for cell cycle-related processes such as cell cycle exit, kinase regulator binding, and ion binding (Fig. 7e). The KEGG pathway enrichment analysis revealed that the DEGs were enriched primarily in pathways related to neuronal development, cytoarchitecture, transdifferentiation, neurotrophin biosynthesis, synaptogenesis, and transmitter biosynthesis (Fig. 7f).

### ATG-derived cells exhibited functional characteristics of typical neurons

**Fig. 8:**
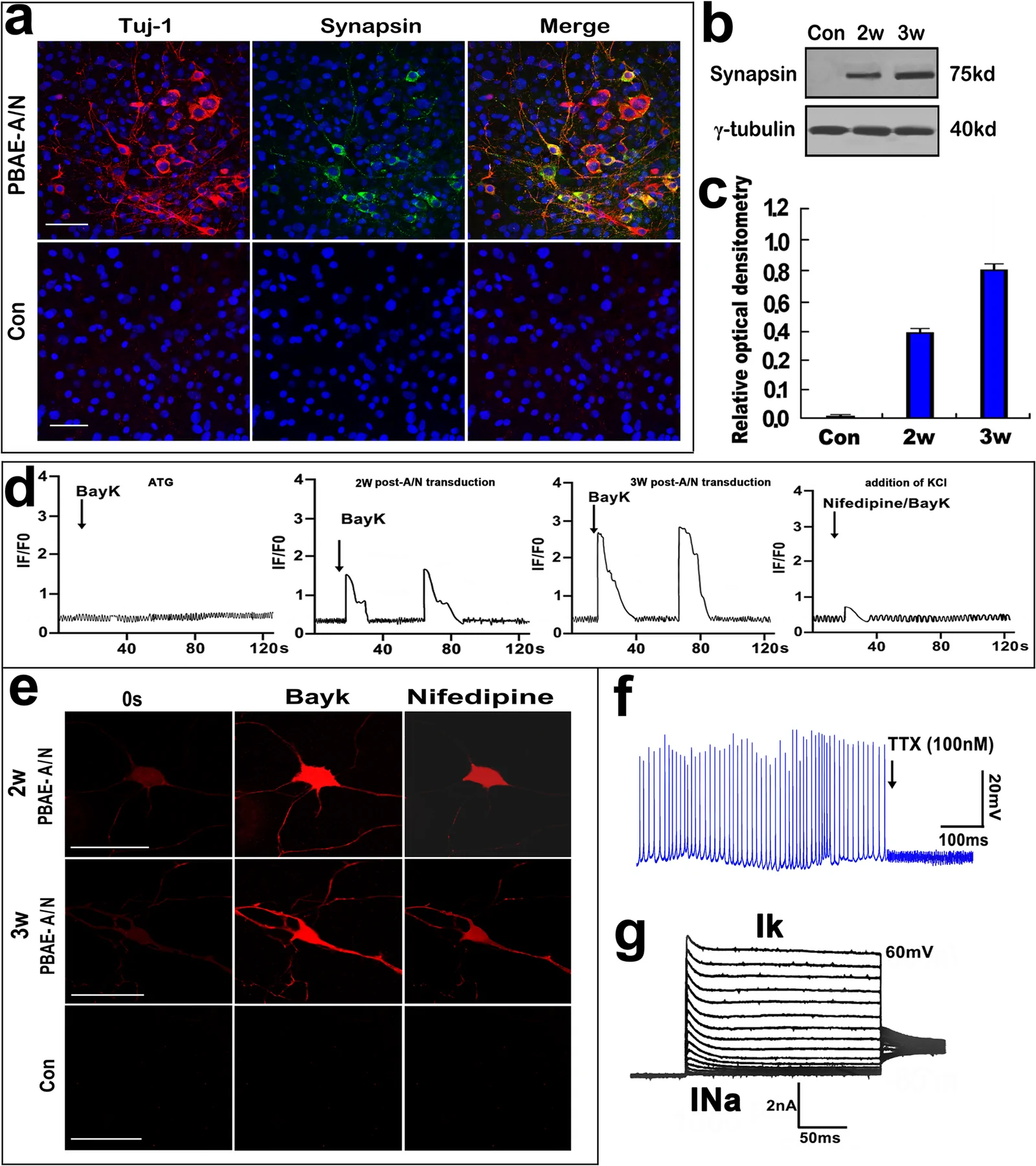
Neurophysiological properties of iNeurons reprogrammed from ATGs. **a** Representative micrographs illustrating the immunolocalization of synapsin in iNeurons derived from ATG at 2 weeks after ASCL1/NGN2 transduction. Scale bars: 100 mm. **b** Western blot analysis of synapsin expression at 2 and 3 weeks after ASCL1/NGN2 transduction, respectively. β-actin served as a loading control. **c** Quantitative analysis of synapsin protein levels detected by Western blotting. **d** Functional assessment of L-type Ca^2+^ channels. The panels show typical calcium responses of ATG-derived iNeurons at 2 and 3 weeks post-induction, respectively. 1 F/F0 indicates the ratio of the fluorescence intensity of cells at 0 s and at the indicated time. BayK (10 mM) was applied with or without nifedipine (5 mM) at the indicated time points. **e** Representative Ca^2+^ images of ATGs or iNeurons derived from ATGs treated with BayK in the presence or absence of nifedipine. Notably, approximately 50 cells were tested per condition; representative images are shown. Scale bar: 50 μm. **f** Current-clamp recording of spontaneous action potentials with varying amplitudes in ATG-reprogrammed neurons. Action potentials were suppressed by TTX. **g** Voltage-clamp recording of ATG-reprogrammed neurons showing sodium and potassium currents evoked by stem depolarization

Although our prior experimental findings have shown that PBAE-A/N transduction drove the reprogramming ATGs to acquire the morphological and biochemical characteristics of wild-type neurons, the functional competence of these reprogrammed neurons (iNeurons) has not been fully characterized. To bridge this knowledge gap, we systematically evaluated synaptic marker expression by immunostaining and Western blot analysis and functionally validated iNeurons through calcium imaging and patch-clamp electrophysiology. First, Tuj-1 and synapsin double-immunostaining revealed that numerous neurons were labeled by punctate synapsin staining, which is indicative of presynaptic puncta formation. No synapsin immunoreactivity was detected in the control group (Fig. 8a). Consistent with the immunostaining data, the result of the Western blot analysis revealed significant synapsin upregulation in reprogrammed cells. Notably, synapsin levels were higher in ATGs transduced with A/N for 2 weeks than those transduced for less than 2 weeks, suggesting the formation of synaptic connectivity in iNeurons (Fig. 8b and c).

To more rigorously characterize the functional properties of the iNeurons, we also performed the calcium imaging of reprogrammed ATGs at 2 and 3 weeks post-A/N transduction. As shown in Fig. 8d, BayK (a calcium channel agonist) treatment elicited robust neuron-specific calcium influx in iNeurons. The increase in fluorescence intensity was approximately 3- to 6-fold greater at 3 weeks post-transduction than at 2 weeks. Notably, the increase in fluorescence persisted after BayK withdrawal and was effectively suppressed by nifedipine (an L-type calcium channel blocker), whereas control ATGs showed no response to BayK stimulation. Intriguingly, the administration of KCl partially restored the calcium fluorescence intensity following nifedipine treatment (Fig. 8d). Morphologically, compared with the cells measured at 2 weeks, the neurons generated from ATG exhibited significantly stronger fluorescence responses at 3 weeks after ASCL1/NGN2 transduction, while control ATGs remained completely nonresponsive, with no detectable fluorescence signal under identical experimental conditions (Fig. 8e), suggesting progressive maturation toward a functional neuronal physiology. Collectively, these findings indicated that these iNeurons acquired the physiological activity of authentic neurons.

To more definitively evaluate whether the iNeurons acquire genuine neuronal electrophysiological properties, we examined whole-cell membrane electrophysiological properties following 3 weeks of reprogramming. As shown in Fig. 8f and g, the majority of iNeurons (7 of 9) exhibited active membrane properties capable of generating repetitive action potentials (APs) upon depolarization. In addition, voltage-clamp recordings revealed fast activation, with inactivating inward currents blocked by tetrodotoxin (TTX), a specific inhibitor of sodium ion channels, demonstrating that these iNeurons possess the characteristics of functional neurons in vitro. Of note, spontaneous APs of varying amplitudes were also elicited, indicating functional neuronal firing patterns. Furthermore, voltage-clamp recordings showed that these iNeurons displayed voltage-sensitive inward and outward currents, which appeared as Na^+^ and K^+^ currents according to their temporal profiles (Fig. 8g).

Collectively, these results indicated that PBAE-A/N-induced iNeurons possess key functional attributes of authentic neurons, including synaptic marker expression, regulated calcium signaling, and active membrane properties enabling AP generation and ion channel activity.

### PBAE-A/N delivery enhances neural functional recovery following SCI

**Fig. 9:**
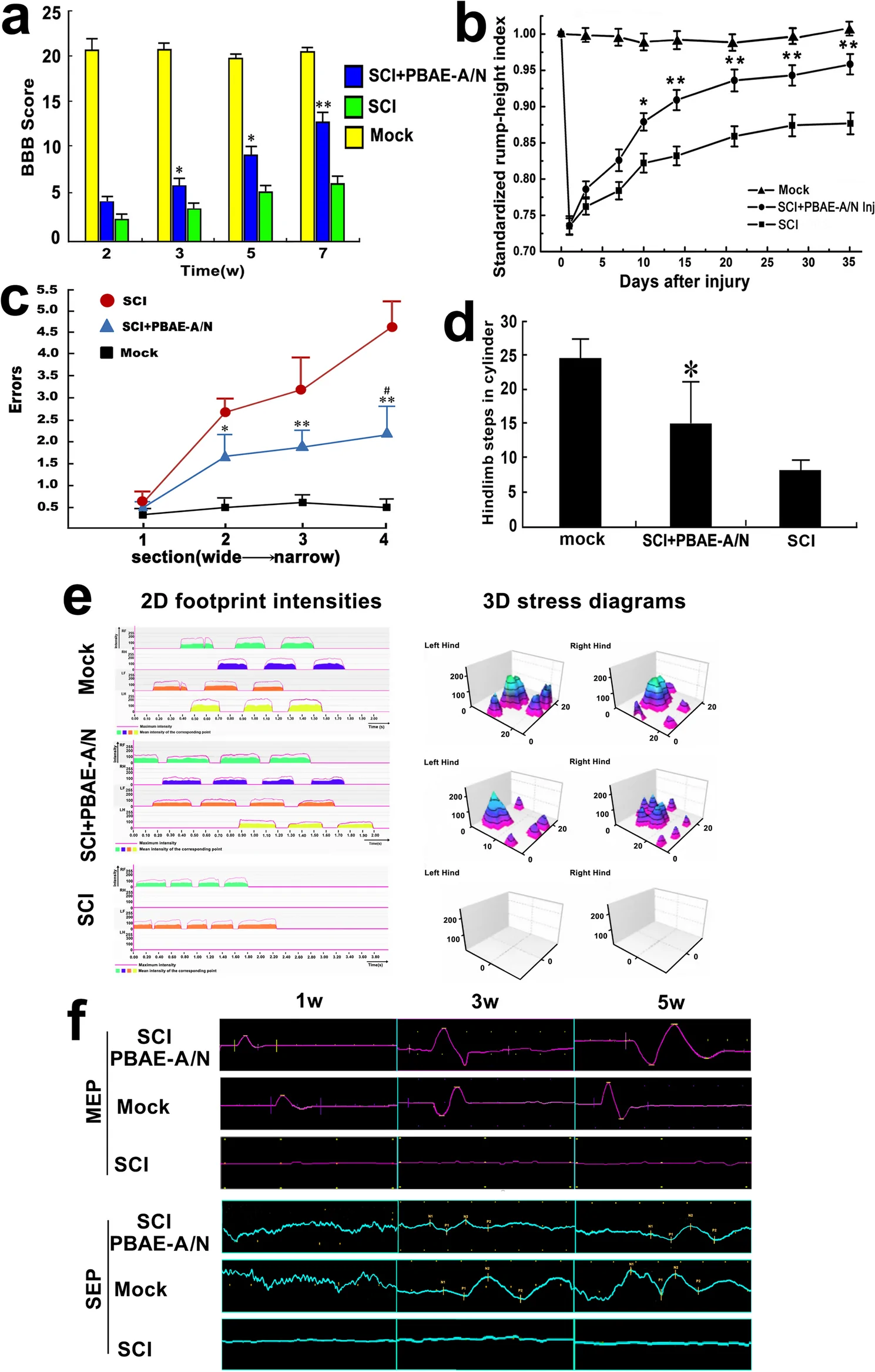
PBAE-A/N administration enhances functional recovery in SCI model rats. **a** BBB locomotor rating scores at various time points following T8–T10 spinal cord compression injury in PBAE-A/N-injected rats. **b** Rump height index (RHI) measurements showing significant improvement in PBAE-A/N-delivered rats at 10, 14, 21, 28, and 35 days post-SCI compared with controls. **c** Beam walking performance (mean errors) across beams of different widths. **d** Hindlimb stepping frequency in the SCI (*n* = 5), PBAE-A/N-delivered (*n* = 5), and sham-operated (*n* = 5) groups was assessed by cylinder test at 5 weeks after operation. **e** Representative diagrams of the 2D and 3D gait analyses performed at 5 weeks after PBAE-A/N delivery, with limb assignments. Left forelimb (LF, orange), right forelimb (RF, green), left hindlimb (LH, yellow), right hindlimb (RH, blue). **f** Temporal changes in MEP and SEP waveform characteristics (latency and amplitude) at 1, 3, and 5 weeks after PBAE-A/N administration. Note that the data are presented as the means ± SEM (*n* = 5, *P < 0.05, **P < 0.01, and ***P < 0.001 by two-way repeated measures ANOVA with post-hoc test)

In addition to characterizing the biochemical and physiological properties of reprogrammed ATGs in vivo and in vitro, behavioral assessments following PBAE-A/N delivery are crucial for comprehensively evaluating their therapeutic potential in preclinical SCI models. As shown in Fig. 9a, BBB scores did not significantly change in the mock-treated control group, whereas PBAE-A/N administration induced a substantial locomotor improvement in SCI animals, with the BBB scores progressively increasing at all four post-treatment time points. The quantitative analysis revealed significantly greater motor recovery in the PBAE-A/N-treated SCI rats than in the untreated SCI control rats (*P < 0.05 and **P < 0.01 at the corresponding time points). Consistent with the BBB locomotor score, the rump height index of PBAE-A/N-injected animals progressively improved and remained significantly higher than that of untreated SCI controls. However, the rump-height indices of the mock-treated controls remained stable throughout the observation period (Fig. 9b). Furthermore, the challenging beam traversal analysis revealed that compared with control rats, SCI-induced rats committed significantly more errors per step than controls. Notably, PBAE-A/N treatment substantially improved motor coordination, as evidenced by progressive reductions in error frequency at 3, 5, and 7 weeks post-treatment (Fig. 9c). Similarly, spontaneous activity testing (in the cylinder test) further showed that SCI rats exhibited significantly impaired hindlimb function, as evidenced by the markedly reduced stepping frequency. This deficit was substantially ameliorated by a targeted PBAE-A/N injection into the GS. The quantitative analysis revealed a statistically significant improvement in PBAE-A/N-treated SCI rats compared with untreated SCI control rats (**p < 0.01), with no significant difference between PBAE-A/N-treated SCI rats and mock-treated control rats, despite a modest reduction in the residual deficit in hindlimb stepping (Fig. 9d).

To quantitatively evaluate neural functional recovery in more detail, we conducted a comprehensive gait analysis using the CatWalk system at 5 weeks after the PBAE-A/N injection (Fig. 9e). Rats in the SCI control group exhibited complete hindlimb paralysis with no weight-bearing capacity, limiting the gait analysis to the forelimb (Fig. 9e, lower left panel). In contrast, PBAE-A/N-treated animals exhibited significant functional recovery, with coordinated movement of both hindlimb albeit slow. While these rats achieved weight-bearing ability, their peak support strength remained significantly reduced (*p* < 0.01) and the contact duration was prolonged (**p < 0.05) compared with those of the sham-operated controls, as clearly shown in the 2D pressure maps (Fig. 9e, left panel). The 3D gait analysis indicated notable improvements in PBAE-A/N-treated rats, with a complete resolution of hindpaw toe curling and near-normalization of plantar contact patterns resembling those of sham-operated animals (Fig. 9e, right panel). However, dynamic pressure measurements indicated subnormal individual toe strike forces. The overall footprint morphology and pressure distribution patterns suggested sustained therapeutic efficacy with a progressive restoration of weight-bearing and locomotion.

Representative MEP and SEP traces (*n* = 10 rats/group), with mean peak latencies and amplitudes, are presented in Fig. 9f. Sham-operated (mock) controls exhibited minimal alterations in MEP and SEP waveforms following surgery. In contrast, compared with sham control rats, SCI rats consistently displayed severe electrophysiological deficits, which manifested as a pronounced reduction in amplitude and significantly prolonged latency, confirming minimal spontaneous recovery. However, the PBAE-A/N-injected groups showed a progressive neurological improvement. The SEP parameters initially recovered as early as 1 week after the intervention, indicating somewhat spontaneous recovery. Notably, at 1 and 3 weeks after PBAE-A/N injection, the animals exhibited comparable evoked potentials, featuring an initial acute decrease in the amplitude followed by gradual recovery. The impairments persisted in these animals compared with those in the mock group (*P < 0.05, **P < 0.01). In contrast, PBAE-A/N-treated animals exhibited an accelerated restoration of SEP amplitudes and conduction velocities and a reduction in latencies. By 5 weeks post-surgery, the amplitudes had approached near-normal levels. A comprehensive waveform analysis revealed that PBAE-A/N treatment significantly improved both MEP and SEP parameters at the 5- and 7- week endpoints compared to untreated SCI control animals. Further detailed changes in SEP and MEP, including the P-wave morphology, are illustrated in Fig. 9f.

### Reprogramming of ATGs into neurons within the glial scar in the lesioned spinal cord

**Fig. 10:**
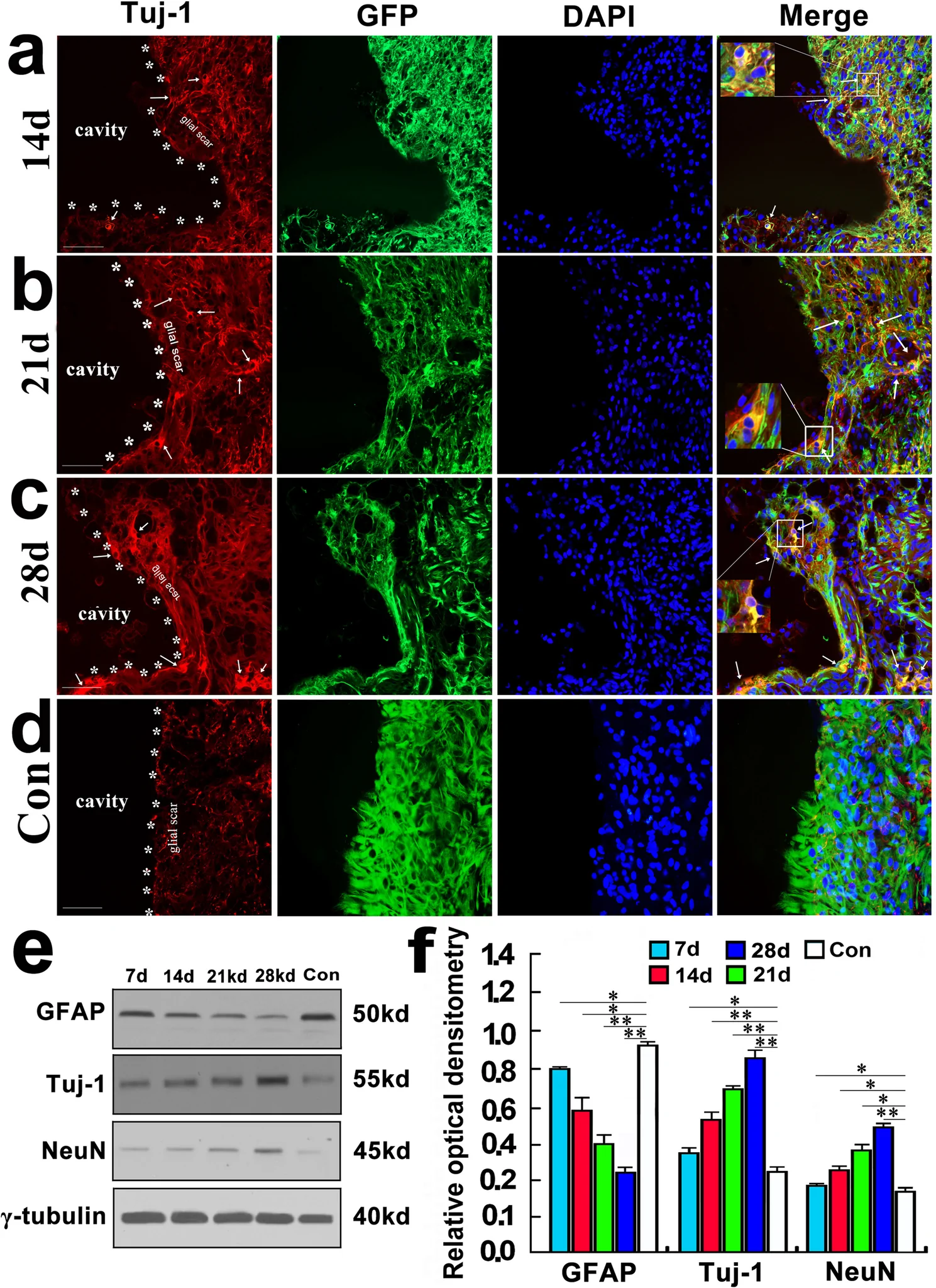
Assessment of the neuronal differentiation potential of PBAE-A/N-Transduced ATGs in vivo. **a**–**c** Image of Tuj-1 immunostaining depicting ATGs within the glial scar in the lesioned spinal cord at 14, 21, and 28 days after the injection of PBAE-A/N, respectively. Notably, the injected PBAE-encapsulated plasmid contained GFAP promoter-driven A/N along with a GFP reporter. Thus, following PBAE-A/N transduction, all GFP^+^ cells are expected to be ATGs, and consequently, Tuj-1^+^ cells should derive from GFP-labeled ATGs. **d** Tuj-1 immunostaining of the glial scar in the lesioned spinal cord after the PBAE-empty vector injection. No Tuj-1 reactivity was observed in GFP^+^ cells. Asterisks (*) mark the glial scar boundary regions; arrowheads (↑) highlight representative Tuj-1^+^ cells colocalized with GFP. The insets show a higher-magnification view of the rectangular region. Scale bar = 100 μm. **e** Western blot analysis of GFAP, Tuj-1, and NeuN expression in the glial scar area at 14, 21, and 28 days after the injection of PBAE-A/N NPs. **f** Quantification of the protein expression levels of GFAP, Tuj-1, and NeuN normalized to that of β-actin. The data are presented as the means ± SEM (*n* = 10; independent experiments *p < 0.05 and **p < 0.01 by two-way ANOVA with a post-hoc test)

To further validate the successful reprogramming of ATGs within the glial scar (GS) of SCI model rats via PBAE-A/N delivery, we performed a targeted in vivo microinjection of PBAE-A/N nanoparticles specifically into the GS region, followed by immunostaining to assess the neuronal conversion. Confocal microscopy of GS sections revealed that at 14 days post-injection (14 DPI), a subset of GFP-labeled cells coexpressed the neuronal marker Tuj-1, although the proportion of double-positive (GFP+/Tuj1+) cells remained relatively low (Fig. 10a). The higher-magnification inset illustrates the morphology of the induced neurons. By 21 and 30 DPI, an increasing number of GFP-labeled cells expressed Tuj-1 and displayed a characteristic neuronal morphology with elaborate neurite outgrowth (Fig. 10b and c). Intriguingly, these dual-labeled cells were predominantly localized at the periphery of the GS, with significantly fewer cells observed in deeper regions. Conversely, no Tuj1-positive cells were observed in the PBAE-empty vector groups (Fig. 10d). These results indicated that forced expression of A/N greatly enhanced the reprogramming of ATGs toward neurons in vivo. To comprehensively evaluate neuronal reprogramming within the GS following PBAE-A/N delivery, we conducted Western blot analyses of GFAP, Tuj-1, and NeuN expression. Consistent with the immunostaining data, the Western blot results also showed a time-dependent increase in Tuj-1 and NeuN expression and a gradual decrease in GFAP levels over time after the injection (Fig. 10e). In contrast, the control group showed no remarkable changes in these neuronal markers (Fig. 10e). The quantitative analysis further confirmed statistically significant differences in the expression of these proteins between the groups (*P < 0.05, **P < 0.01; Fig. 10f). Collectively, these findings provide compelling in vivo evidence that PBAE-A/N delivery effectively reprogrammed ATGs within the GS into neurons.

### Endogenous activation of pro-neurogenic TFs and modulators during ATG reprogramming into neurons

**Fig. 11:**
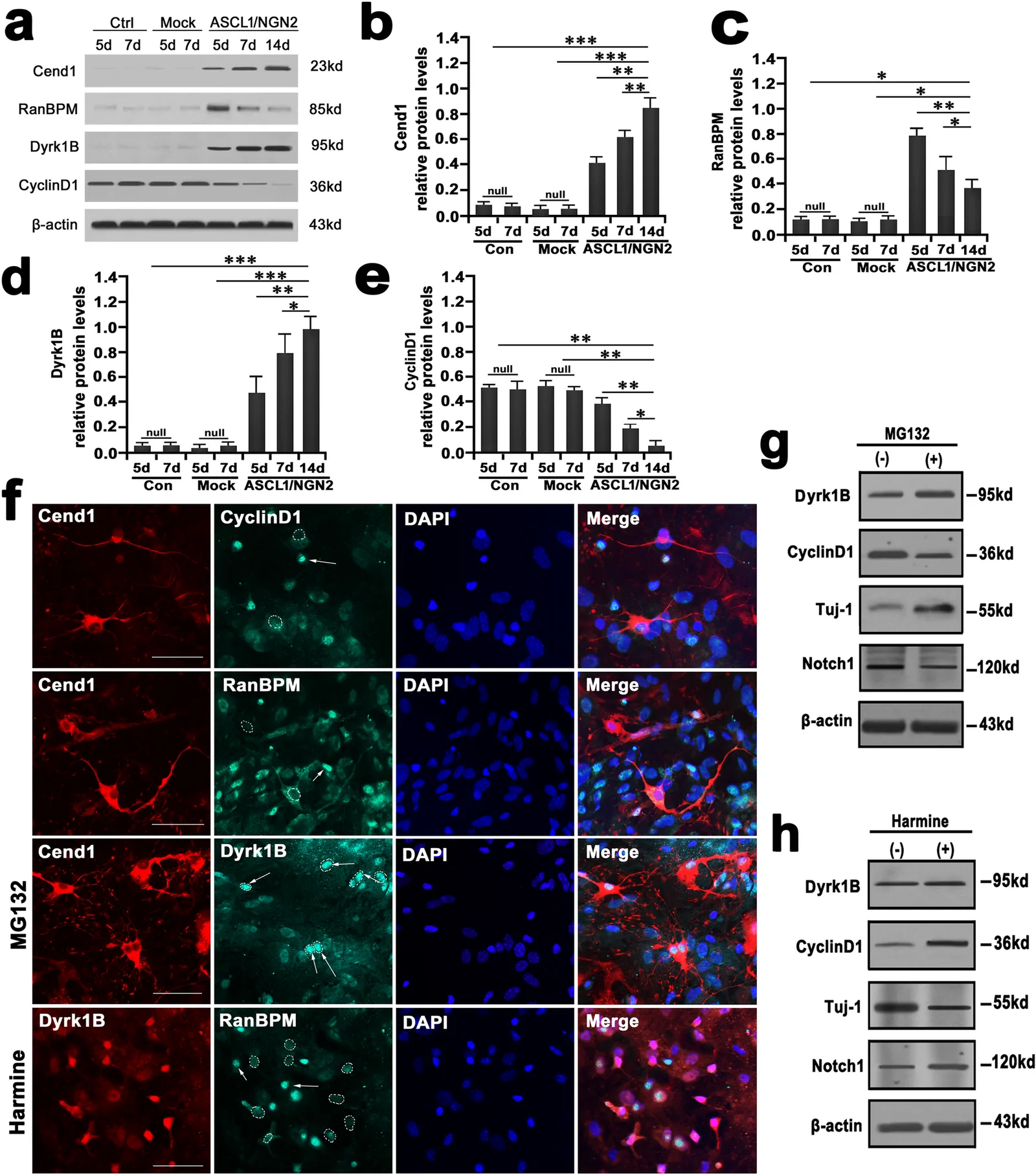
Possible molecular mechanism orchestrating ATG-to-neuron reprogramming. **a** Representative Western blot bands showing the expression levels of Cend1, RanBPM, Dyrk1B, and Cyclin D1 in PBAE-A/N-transduced ATGs at 5–14 days. **b**–**e** Quantitative analysis of the levels of these molecules normalized to those of β-actin. (*n* = 3/group). The data are presented as the means ± SEM (*n* = 3 independent experiments *P < 0.05, **p < 0.01, and ***p < 0.001 by one-way ANOVA with a post-hoc test). **f** Immunostaining showing Cend1 expression in PBAE-A/N-transduced ATGs under the indicated conditions (treatment with MG132, a selective 26 S proteasome inhibitor targeting Dyrk1B, or harmine, a specific kinase inhibitor of the Dyrk1 family), depicting the effect of Dyrk1B on enhancing iNeurons generation and maturation. Scale bars = 150 μm. **g** Representative Western blot bands showing the expression levels of Dyrk1B, Cyclin D1, Tuj-1, and Notch1 in PBAE-A/N-transduced ATGs cultured in the presence of MG132 (a selective 26 S proteasome inhibitor targeting Dyrk1B). β-actin served as a loading control. **h** Representative Western blot bands showing the levels of Dyrk1B, Cyclin D1, Tuj-1, and Notch1 expression in PBAE-A/N-transduced ATGs cultured in the presence of harmine. β-actin served as a loading control

The KEGG clustering analysis revealed that peak-associated genes were significantly enriched in the processes related to neurogenesis, neuronal development, brain development, gliogenesis, and glial cell development (Supplementary Fig. 1), which is consistent with the observed neuronal features. Similarly, RNA-seq data indicated that compared with ATGs, multiple members of these TF gene families are upregulated or highly expressed in iNeurons and neurons (Supplementary Fig. 2). To further elucidate the molecular mechanisms underlying A/N-mediated ATG-to-iNeuron reprogramming, we conducted immunoblotting analyses to examine the dynamic changes in the expression of critical TFs. Strikingly, on days 5, 7, and 10 after A/N transduction and induction treatment, the levels of Cend1, its binding partner RanBPM1, dual-specificity tyrosine-phosphorylation-regulated kinase 1B (Dyrk1B), and Cyclin D1 exhibited significant time-dependent alterations in A/N-treated ATGs. Specifically, Cend1 and Dyrk1B expression was markedly upregulated, whereas RanBPM1 and cyclinD1 expression progressively decreased during the reprogramming of ATGs into iNeurons. Notably, the aforementioned molecules were undetectable in both the untreated control ATG and mock-transduced ATG groups (Fig. 11a). A quantitative analysis of Cend1, RanBPM, Dyrk1B, and Cyclin D1 expression in A/N-transduced ATGs revealed significant temporal differences compared with their counterparts across different time points, whereas no notable differences were observed between untreated ATGs and mock-transduced controls (Fig. 11b–e). These findings were corroborated by immunostaining and confocal microscopy at 14 days after A/N transduction. All Cend1-positive cells exhibited a neuronal morphology and minimal or no Cyclin D1 or RanBPM reactivity (Fig. 11f, upper 2 panels). Notably, Cend1-positive cells significantly enhanced Dyrk1B reactivity in the presence of MG132 (a 26 S proteasome inhibitor that specifically prevents tyrosine-phosphorylated protein degradation). Conversely, treatment with harmine (a high-affinity kinase inhibitor of Dyrk family members) resulted in substantially reduced or absent RanBPM reactivity in Dyrk1B-positive cells, suggesting that RanBPM dually modulates Dyrk1B by suppressing its kinase activity while promoting its degradation. These results indicated that Cend1 could prevent RanBPM-dependent Dyrk1B degradation.

Although the abovementioned data have shown the upregulation of the Cend1 signaling cascade is involved in astrocyte-to-neuron reprogramming events, the functional significance of this pathway remains unclear. Therefore, we further assessed the expression of neuronal markers in A/N-transduced ATGs following treatment with pathway-specific inhibitors or activators. Intriguingly, treatment with MG132 or harmine in different combinations dramatically altered the ATG reprogramming efficiency by approximately 3- to 5-fold. Specifically, MG132 reduced the expression of Cyclin D1 and Notch1 while increasing Dyrk1B and Tuj-1 levels (Fig. 11g). Conversely, harmine treatment markedly increased Cyclin D1 and Notch1 expression (3-fold to more than 6-fold) and suppressed Dyrk1B and Tuj-1 expression (Fig. 11h). These findings provide compelling evidence that the Cend1/RanBPM/Dyrk1B signaling axis plays a pivotal role in orchestrating ATG-to-neuron reprogramming.

## Discussion

Spinal cord Injury (SCI) is a devastating CNS trauma with exceptionally high morbidity and mortality, and it represents an intractable global challenge in neurotrauma medicine [65]. Due to the unique cytoarchitecture of the spinal cord [66], hitherto, no curative treatments are available for SCI, particularly in severe cases. Notwithstanding current conventional therapies provide a limited amelioration of neurological dysfunction following SCI [67], these therapeutic applications still fail to counteract neuronal loss-induced circuitry disruption [68], apart from eliciting onlymodest neuronal activation in damaged regions with negligible functional recovery [69]. Critically, emerging evidence indicates that progressive postinjury microenvironmental deterioration driven by multifactorial pathology, including extensive neuronal apoptosis/necrosis [70] and glial scar formation as critical regeneration barriers [31, 71], severely impedes neural repair. Therefore, strategic glial scar ablation combined with neuronal replacement may hold promise for guiding neural regeneration and integrating newly replenished neurons within the host neural circuits in the lesioned area [39, 72], ultimately restoring neural function.

In this study, we present in vitro and in vivo evidence for an efficient nonviral strategy to promote neural regeneration by reprogramming endogenous ATGs within the glial scar (GS) into functional neurons, concurrently resolving the dual challenges of neuronal loss and GS barriers in CNS repair. Our approach utilizes biodegradable PBAE nanoparticle-loaded ASCL1/NGN2 plasmids, representing a significant advancement over viral vector methods. This approach mitigates the risk of insertional mutagenesis and immunogenicity while enabling precise, localized gene editing. Our results show that ASCL1/NGN2-mediated reprogramming not only efficiently generates neurons in situ but also actively remodels GS structures. Notably, the rapid downregulation of GFAP expression and the scar remodeling within 1–3 weeks post-transduction progressively eliminate a major impediment to regeneration, whereas the concurrent upregulation of Tuj-1 and MAP2 expression substantiates neuronal conversion. The induced neurons (iNeurons) closely resemble genuine neurons in several key aspects: exhibiting typical neuronal morphological attributes (including the soma and robust neurite extension), sharing biochemical phenotypic features, and displaying matching global gene expression profiles. Comprehensive functional analyses indicated that nearly all converted cells adopted in vitro identities and functions of authentic neurons, including specialized neuronal Ca2 + influx, synaptic activity, action potentials, and voltage-gated sodium (INa) and potassium (Ik) currents. Critically, the delivery of PBAE nanoparticles loaded with these TFs to the GS of the injured spinal cord alleviated neurological deficits, further validating the effectiveness of the reprogramming method. To our knowledge, this report is the first highlighting the generation of functional neurons from GS astroglial cells and the simultaneous GS remodeling after SCI via PBAE-mediated TF delivery. This strategy effectively eliminates the GS mechanochemical barrier through ATG reprogramming, creating a favorable microenvironment for neural regeneration and functional recovery. This approach circumvents the intrinsic risks associated with conventional viral vectors and the complexity of small-molecule protocols, proving highly efficient at reprogramming of ATGs into functional iNeurons both in vitro and in vivo. Furthermore, direct conversion without lineage switching validated the establishment of neural functional specificity. Thus, these dual-pronged effects (neuronal replenishment coupled with barrier removal) provide a promising solution for the critical unmet need in CNS injury therapy.

In previous studies, TF-mediated cell reprogramming approaches have typically been favored and widely adopted because of their high efficiency and relatively simple implementation process [73]. Although alternative methods, such as the use of cytokines, chemical small molecules, or other specific conditional interventions, to directly convert somatic cells into neurons are highly important for lineage reprogramming, this process ultimately requires intrinsic epigenetic alterations [74]. Critically, these essential epigenetic modifications are tightly linked to the targeted regulation provided by TFs [75]. Therefore, specific TFs known to function as fate-determinants in neurogenesis and neural development are often selected to drive epigenetic reprogramming, enabling the conversion of glial cells such as ATGs into neurons [76]. To date, several studies have achieved neural reprogramming by introducing neural-specific TFs into glial cells or other somatic cells. For instance, Berninger et al. [77] converted cultured ATGs from the early postnatal cerebral cortex into functional neurons through forced expression of *Ascl1* and *Ngn2*. This work highlights the key roles of these TFs in promoting neural development and nervous system regeneration and enhancing reprogramming efficiency. Similarly, Grande et al. [78] reprogrammed ATGs from the adult neocortex and striatum into glutamatergic and GABAergic neurons by overexpressing Ngn2 in combination with treatment of FGF-2 and EGFs. Here, ASCL1 acts as an efficient pioneer pro-neurogenic transcription factor that initiates the complex molecular conversion of diverse cell types into neurons, while Ngn2 serves as both an enhancer of neuronal reprogramming efficiency and a modulator of neuronal subtype specification. Additionally, NGN2 plays a key role in redirecting regulatory priorities toward the positive regulation of nervous system development, neuronal maturation and synaptic integration, and axonal guidance. Given the critical role of individual TFs in cellular reprogramming [79], successful reprogramming of ATGs into induced neurons (iNeurons) via *Ascl1* and *Ngn2* transduction in combination with defined factor treatment may prove more efficient than existing strategies. This approach could substantially pave the way for novel drug therapies targeting SCI.

In this study, we first established an in vitro astroglial wound model in cultured ATGs using a mechanical scratch-wound assay to validate our hypothesis and accurately determine the reprogramming efficiency of ATGs within GS into neurons. Our analysis revealed marked hypertrophy, proliferation and cytoplasmic extension of ATGs at the scratch margin after the scratch injury, followed by significant extension of hypertrophic processes into the denuded area over time, and an increasing number of ATGs at the scratch margin exhibited time-dependent increase in the expression of CSPG and Brevican. Furthermore, CSPG- and Brevican-positive cells appeared diffusely in regions distant from the scratch. Concurrently, the number of BrdU-positive cells gradually increased. These results strongly support the successful establishment of the glial scar model. Therefore, these converted iNeurons can be confirmed to originate solely from glial scar-resident ATGs without contributions from contaminating cells. Critically, our work demonstrated the feasibility of using PBAE-loaded proneural TFs *NGN2* and *ASCL1* to generate iNeurons for cell replacement therapy for SCI. This approach offers the following two key advantages: First, This combination leverages the complementary advantages of both TFs, which possess the substantial ability to not only reprogram rodent ATGs into induced neurons but also to remodel the mechanochemical barrier formed by the GS. This is primarily attributed to the fact that the specific transduction of two TFs into ATGs relies upon a rat GFAP-driven promoter subcloned and inserted into the plasmid, thus providing a favorable microenvironment for neural regeneration and functional reconstruction. Second, poly(β-amino ester) (PBAE), a biodegradable biomaterial with low cytotoxicity, is used as a nonviral vector for *ASCL1/NGN2* gene delivery due to their high DNA/RNA loading capacity and ease of self-assembly into nanoparticles. By optimizing the PBAE-to-DNAratio, we found that the biomaterial has a high capacity to carry genes. Given that the stability of PBAE nanoparticles arises from the synergistic interplay of polymer structure, preparation process, and environmental conditions. Rational polymer structural design (e.g., incorporating hydrophobic segments or PEGylation) and optimizing preparation parameters (e.g., N/P ratio and microfluidic conditions), and their stability in physiological environments can be markedly enhanced despite inherent susceptibility to degradation. In DNA release experiments, Our results indicate that the nanoparticles remained stable for over 48 h, as confirmed by hydrodynamic diameter and polydispersity index measurements. PBAE degradation is highly tunable, governed by a complex interplay of multiple interrelated factors, including pH, temperature, and chemical structure, and ranges from rapid disintegration within tens of minutes to slow degradation lasting several months. For our designed PBAE formulation, complete intracellular degradation occurs in approximately 4 days. Furthermore, the CCK-8 analysis revealed minimal cytotoxicity at optimal ratios, indicating that PBAE is a safe and highly efficient nucleic acid carrier. Notably, iNeurons generated by PBAE-ASCL1/NGN2-mediated reprogramming of ATGs exhibited authentic neuronal morphological and biochemical properties. The introduction of PBAE-A/N into ATGs significantly increased the conversion efficiency, as evidenced by accelerated morphological changes, neuronal marker expression, reduced proliferation, and cell cycle exit during reprogramming. Concurrently, numerous characteristics of ATGs and the GS progressively diminished over time. Interestingly, ASCL1/NGN2 transduction also upregulated the expression of key proneural TFs (Cend1, Transgelin3, and Brn3a) and the neuronal subtype marker CHAT. Cend1 plays a dual role in neurogenesis and neuronal fate specification by dynamically regulating mitochondrial fusion/fission [80] and promoting the cell cycle exit and activation of a proneuronal gene network to suppress cell proliferation through the p53/Cyclin D1/pRb pathway [81]. Transgelin-3, an actin-binding protein family, can actively promote neuronal differentiation, migration, axon guidance, and synapse formation during neural development [82–84]. Consistently, a latest study also revealed the importance of Brn3A in sensory neuron differentiation/survival [85], neuronal subtype specification [86], and axon growth/target innervation [87]. Although NeuroD1 is a potent CNS injury reprogramming factor that converts > 90% of reactive ATGs into functional neuron-like cells [88], it faces critical limitations: (i) induced neurons are predominantly glutamatergic, consistent with its developmental subtype specification role [89]; (ii) it effectively reprograms gray matter astrocytes but shows minimal efficacy on resistant white matter ATGs; and (iii) its overexpression upregulates cleaved caspase-3, indicating pro-apoptotic risks. In contrast, the ASCL1 + NGN2 combination exhibits a superior safety profile. Based on these findings [76, 77], we speculated that *ASCL1/NGN2* play pivotal roles in orchestrating the efficient neuronal reprogramming of ATGs and driving neuronal commitment.

To further elaborate on the cellular conversion, we analyzed global transcriptome profiles and the expression of key proneurogenic genes before and after ATG reprogramming. Hierarchical clustering analysis and pairwise scatter plots revealed that iNeurons (PBAE-A/N-transduced ATGs) shared highly similar gene expression patterns to wild-type neuron but differed significantly from both untransduced ATGs and PBAE-empty vector-transduced ATGs. The expression of genes more related to neurogenesis and neuronal commitment increased in iNeurons, whereas the expression of genes associated with ATG identity and cell cycle maintenance was downregulated, corroborating the observed morphological and phenotypic characteristics. Bioinformatic analysis revealed that the DEGs were enriched in signaling pathways with widespread roles in cell growth, neural differentiation, neurite extension/axon guidance, synapse formation, and protein translation. Notably, the neurotrophin signaling pathway, which is critical for neuronal development [90, 91], was enriched in A/N-transduced ATGs. Similarly, the Wnt signaling pathway, known for its role in the differentiation of neural progenitor cells [92, 93], was markedly enriched. The PI3K-AKT and ErbB signaling pathways, which are important for lineage specification during neural differentiation and cell survival during the early stages of neuronal development [94, 95], were also enriched. The enriched signaling pathways were detected in PBAE-A/N-transduced ATGs and progressively increased over time, suggesting that these signaling molecules are essential for driving ATG-to-neuron reprogramming mediated by A/N transduction combined with neural induction.

In addition to characterizing the morphology, biochemical phenotype, and whole-genome transcriptional profiles of iNeurons derived from ATGs, we rigorously validated their neuronal functionality through comprehensive in vitro and in vivo experiments. After culture under defined culture conditions in vitro following PBAE-N/A nanoparticle transduction, the converted ATGs exhibited hallmark neuronal functions, including synapse formation, neuron-specific calcium signaling, and electrophysiological activity. Critically, within the GS of injured spinal cord models injected with PBAE-A/N nanoparticles, ATGs significantly expressed the neuronal marker Tuj-1 at various time points post-injection. Notably, the observed dual-labeled GPF+/Tuj-1 + cells within the GS unequivocally originated from reprogrammed ATGs as the injected PBAE-encapsulated a plasmid containing *GFAP* promoter-driven ASCL1 and NGN2 expression alongside a GFP reporter. Strikingly, this in situ conversion of ATGs to iNeurons significantly ameliorated SCI symptoms and enhanced functional recovery to varying degrees. Behavioral assessments (beam walking and 2D/3D gait analyses) and evoked potential recordings (MEP/SEP amplitude and frequency) showed progressive improvements over 3 weeks after the injection, approaching the levels observed in the normal control groups. This functional recovery is primarily attributed to two key mechanisms: the reprogrammed ATGs compensate for neuronal loss by integrating into host neural circuits, and they reduce the inhibitory GS barriers, thereby facilitating the neuroplasticity of existing endogenous axonal networks. The observed in situ neurogenesis marked by Tuj-1 + cells within the GS, further supports this mechanism, although improvements across different behavioral indices exhibited temporal heterogeneity. Collectively, our results indicate the significant therapeutic efficacy of PBAE-A/N nanoparticle delivery for functional recovery in a rodent SCI model.

Finally, given the complexity of in situ ATG reprogramming, elucidating the underlying mechanism remains considerably important. In addition to the previously identified modulators responsible for ATG conversion, Ascl1/Ngn2 transduction combined with defined factor treatment upregulated *Cend1* and *Dyrk1B* but downregulated *RanBPM* and *Cyclin D1*. This coordinated shift in the expression pattern implies that these molecules participate in the reprogramming process. Critically, *Cyclin D1* downregulation rapidly suppressed the G1-to-S phase transition, inducing cell cycle arrest, a critical finding confirmed by significantly reduced Cyclin D1 levels [96, 97]. Cend1, a neuronal lineage-specific regulator with dual roles in neurogenesis and neuronal fate specification [98, 99], was activated during the conversion of ATGs. It promotes cell cycle exit and mitochondrial dynamics by suppressing proliferation via the P53/cyclin D1/pRb pathway [98] while activating a pro-neuronal gene network [100]. Notably, *Cend1* overexpression drives progenitor cell differentiation by suppressing Cyclin D1 and activating the P53/pRb pathway [99, 101]. More strikingly, in our system, *Cend1* expression was markedly upregulated in PBAE-A/N-transduced ATGs, consistent with the suppression of CyclinD1 expression, further suggesting that Cend1 is involved in ATG reprogramming into neurons. Of relevance to our present findings, *RanBPM* has been recently implicated in cell cycle progression in precursors, and its upregulation directly affects the ability of Dyrk1B to target cyclin D1 [102, 103]. In our study, compared with mock and control ATGs, PBAE-A/N-transduced ATGs treated with FSK exhibited progressive increases in Dyrk1B/Cend1 expression and decreases in RanBPM/Cyclin D1 expression (observed using immunoblotting and immunostaining). Given that Dyrk1B, a kind of RanBPM partner, negatively regulates *Cyclin D1* [104] and functionally intersects with Cend1/RanBPM [105], it plays a critical role in orchestrating ATG reprogramming initiation and progression. To substantiate these finding, we used MG132, a selective 26 S proteasome inhibitor that targets Dyrk1B [106], or harmine, a specific inhibitor of the Dyrk1 family [107], to inhibit the abovementioned signaling molecules essential for cell cycle exit, epigenetic regulation, cell proliferation, and cell fate commitment. Intriguingly, treatment with MG132 during PBAE-A/N-induced reprogramming drastically reduced *CyclinD1* and *Notch1* expression while increasing Dyrk1 and Tuj-1 expression. Harmine reversed these changes (Figs. 11g and h), confirming its pathway specificity. These results further demonstrate that this dynamic regulation of the Cend1/RanBPM/Dyrk1/Notch1/CyclinD1 axis is necessary, but not sufficient for astrocyte-to-neuron conversion. Based on these findings, we speculate that ASCL1/NGN2 orchestrate ATGs-to-neuron conversion via collaborative Cend1/RanBPM/Dyrk1-mediated downstream signaling and crosstalk with the Notch1/Cyclin D1 pathway.

## Conclusions

In summary, we established a promising nonviral strategy for in situ reprogramming of ATGs within the GS region into neurons, while simultaneously reducing the inhibitory GS. This approach significantly enhances neural regeneration and functional recovery. The delivery of the ASCL1 and NGN2 genes via PBAE cationic nanoparticles resulted in low cytotoxicity and high transfection efficiency, effectively mitigating the risks associated with viral vectors, such as insertional mutagenesis and tumorigenesis. Furthermore, the reprogrammed ATGs acquired a typical neuronal morphology and biochemical phenotype. Crucially, these converted cells exhibited in vitro identities and functionalities indistinguishable from those of authentic neurons. More importantly, the delivery of PBAE nanoparticles loaded with these TFs to the injured spinal cord GS alleviated neurological deficits. To the best of our knowledge, this report is the first describing a therapeutic system that simultaneously addresses dual challenges of neuronal loss and GS barriers for CNS injury repair through in situ ATG reprogramming. Although the detailed molecular mechanism underlying ASCL1/NGN2-triggered ATG reprogramming into neurons requires further investigation, our results reveal that the reprogramming process orchestrates downstream signals mediated by *Cend1*,* RanBPM*,* and Dyrk1*, particularly through crosstalk with the Notch1/Cyclin D1 pathway. This collaborative signaling network drives epigenetic silencing of the *GFAP* gene and neuronal commitment. The efficient and reliable reprogramming approach holds promise as an alternative therapeutic strategy for achieving neural regeneration and functional recovery after SCI through autologous cell-based replacement.

## Supplementary Information

Supplementary Material 1. Fig S1. KEGG analysis of differential genes relative to neurogenesis and cell reprogramming. Fig S2. Gene ontology (GO) enrichment analysis of upregulated genes associated with KEGG pathway between primary neurons, induced neurons transduced with ASCL1/NGN2, and ATGs. (a) upregulated KEGG pathway genes between ATGs and induced neurons (transduced with PBAE-ASCL1/NGN2 for 14 d), (b) Upregulated KEGG pathway genes between primary neurons and induced neurons (transduced with PBAE-ASCL1/NGN2 for 14 d). (c) Upregulated KEGG pathway genes between ATGs and induced neurons (transduced with PBAE-ASCL1/NGN2 for 21 d). (d) Upregulated KEGG pathway genes between primary neurons and induced neurons (transduced with PBAE-ASCL1/NGN2 for 21 d). Table S1 Primers used for reverse transcription polymerase chain reaction. Table S2 Antibody information.

## Data Availability

The data that support the ﬁndings of this study are available from the corresponding author upon reasonable request.

## Abbreviations

PBAE: Poly(beta-amino ester)
TFs: Transcription factors
ASCL1: Achaete-scute homolog 1
NGN2: Neurogenin 2
NPs: Nanoparticles
GSs: Glial scars
CNS: Central nervous system
ATG: Astroglia
Cend1: Cell cycle exit and neuronal differentiation 1
RanBPM: Ran-binding protein in the microtubule-organizing center
Dyrk1: Dual-specificity tyrosine-regulated kinase 1
GFAP: Glial Fibrillary Acidic Protein
Tuj-1: Beta-tubulin III
MAP2: Microtubule-associated protein 2
Notch1: Neurogenic locus notch homolog protein 1
Cyclin D1: G1/S-specific cyclin-D1
SB431542: 4-[4-(1,3-Benzodioxol-5-yl)-5-(2-pyridinyl)-1H-imidazol-2-yl]-benzamide
FSK: Forskolin
VPA: Valproic acid
CSPG: Chondroitin sulfate proteoglycan
DCX: Doublecortin
BrdU: Bromodeoxyuridine
SYN: Synapsin
NeuN: Neuronal Nuclei
Brn3a: Brain-specific homeobox/POU domain protein 3A
CHAT: Choline Acetyltransferase
EDU: 5-Ethynyl-2'-deoxyuridine
BBB: Basso, Beattie, and Bresnahan
RHI: Rump-height index
PBAE-A/N NPs: Poly (beta-amino ester)-loaded ASCL1/NGN2 plasmid nanoparticles
