# Supplementary material for: PBAE nanoparticle-mediated delivery of ASCL1 and NGN2 genes for astroglia-to-neuron reprogramming to remodel glial scar for spinal cord injury repair
Source: Stem Cell Res Ther. 2026 Jul 4;17:335. doi: 10.1186/s13287-026-05159-2 (PMC13613702; doi:10.1186/s13287-026-05159-2)
Supplement: Supplementary file 1 [file 13287_2026_5159_MOESM1_ESM.zip › ARRIVE Essential 10 Checklist.pdf]

ARRIVE Essential 10 Checklist

Item	Recommendation	Section/line number, or reason for not reporting
**Study design**	1. For each experiment, provide brief details of study design including:<br>a. The groups being compared, including control groups. If no control group has been used, the rationale should be stated.<br>b. The experimental unit (e.g. a single animal, litter, or cage of animals).	Materials and Methods, paragraph 2 at pages 14<br>SD rats were divided into three groups: normal control (no SCI), SCI+delivery of PBAE NP-loaded empty plasmid, and SCI + delivery of PBAE NP-loaded A/N (n = 10 per group).
**Sample size**	2. a. Specify the exact number of experimental units allocated to each group, and the total number in each experiment. Also indicate the total number of animals used.<br>b. Explain how the sample size was decided. Provide details of any a priori sample size calculation, if done.	Materials and Methods, paragraph 2 at pages 14<br>a. A total of 30 rats were used (n = 10 per group).<br>b. Sample size was determined based on prior animal experiments and to ensure sufficient statistical power (80%) to detect a 30% difference between groups at α = 0.05.
**Inclusion and exclusion criteria**	3. a. Describe any criteria used for including and excluding animals (or experimental units) during the experiment, and data points during the analysis. Specify if these criteria were established a priori. If no criteria were set, state this explicitly.<br>b. For each experimental group, report any animals, experimental units or data points not included in the analysis and explain why. If there were no exclusions, state so.<br>c. For each analysis, report the exact value of n in each experimental group	Materials and Methods, paragraph 1 at pages 10, paragraph 1 at pages15, paragraph 2 at pages 18, respectively.<br>a. Inclusion criteria: All rats that survived surgery and completed the study were included. No exclusion criteria were pre-established.<br>b. Only animals with stable or pronounced neurological deficits were included in the behavioral tests. No animals or data points were excluded from the analysis.<br>c. For behavioral analysis, n = 5 per group; For SEP or MEP recordings, n=10; for histology, n =3 per group.
**Randomisation**	4. a. State whether randomisation was used to allocate experimental units to control and treatment groups. If done, provide the method used to generate the randomisation sequence.<br>b. Describe the strategy used to minimise potential confounders such as the order of treatments and measurements, or	Materials and Methods, paragraph 1 at pages 10, paragraph 1 at pages15, paragraph 2 at pages 18, respectively.<br>a. Randomization was achieved by means of a random number table generated in Microsoft Excel.<br>b. The order of treatment administration and the placement of cages on the rack

	animal/cage location. If confounders were not controlled, state this explicitly.	were randomized to mitigate potential location effects.
**Blinding**	5. Describe who was aware of the group allocation at the different stages of the experiment (during the allocation, the conduct of the experiment, the outcome assessment, and the data analysis).	Materials and Methods, paragraph 1 at pages15, paragraph 2 at pages 18. Allocation was performed by an investigator not involved in subsequent experiments. Surgeries, behavioral assessments, and functional scores were conducted by experimenters blinded to experimental group allocation. Data analysis was also performed under blinded conditions.
Outcome measures	6. a. Clearly define all outcome measures assessed (e.g. cell death, molecular markers, or behavioural changes).<br>b. For hypothesis-testing studies, specify the primary outcome measure, i.e. the outcome measure that was used to determine the sample size.	Materials and Methods, Sections 2, pages 10–19<br>a. Outcome measures included: (1) ATG proliferation within SC quantified by BrdU+ cell counting, (2) ATG-to-neuron reprogramming quantified by Tuj-1+ cell counting, (3) cell proliferation during ATG-to-neuron reprogramming quantified by EdU+ cell counting, (4) the expression levels of neuronal markers assessed by optical densitometry assay, (5) behavioural changes and neural function recovery assessed by BBB score, RHJ, Error times HSC, and footprint intensity, (6) SYN expression quantified by optical densitometry assay, and (7) the expression levels of pro-neurogenic TFs and modulators during ATG reprogramming into neurons measured by optical densitometry assay.<br>b. The primary outcomes measure was BBB score, RHJ, Error times HSC, and footprint intensity, which was used to estimate sample size.
**Statistical methods**	7. a. Provide details of the statistical methods used for each analysis, including software used.<br>b. Describe any methods used to assess whether the data met the assumptions of the statistical approach, and what was done if the assumptions were not met.	Materials and Methods, paragraph 2 at pages19<br>a. Statistical analyses were conducted using SPSS 10.0. For two group comparisons, Student's t-test or Kolmogorov-Smirnov test were used for statistical analysis.<br>b. For comparisons among multiple groups under different conditions, one-way ANOVA

		was employed. A P-value of less than 0.05 was considered statistically significant. If assumptions were violated, nonparametric tests (Mann–Whitney U or Kruskal–Wallis) were used.
**Experimental animals**	8. a. Provide species-appropriate details of the animals used, including species, strain and substrain, sex, age or developmental stage, and, if relevant, weight.<br>b. Provide further relevant information on the provenance of animals, health/immune status, genetic modification status, genotype, and any previous procedures.	**Materials and Methods,** paragraph 2 at page 5, paragraph 1 at page 6, and paragraph 2 at page 14<br>a. Embryonic day 15 Sprague–Dawley (SD) rats were used for primary culture in this study.<br>b. Adult (SD) rats (2.5 months old, 200-250 g) were used for animal models and behavioral in this study.<br>b. Rats were obtained from the Xi'an Jiaotong University and housed under specific pathogen-free conditions with a 12 h light/dark cycle and free access to food and water. No genetic modifications or prior procedures were applied.
**Experimental procedures**	9. For each experimental group, including controls, describe the procedures in enough detail to allow others to replicate them, including:<br>a. What was done, how it was done and what was used.<br>b. When and how often.<br>c. Where (including detail of any acclimatisation periods).<br>d. Why (provide rationale for procedures).	Materials and Methods, paragraph 2 at page 14 paragraph 2 at page 18<br>a. Spinal cord injury was developed using a modified Allen method.<br>b. Injections of PBAE NP-loaded A/N plasmids were performed one week after SCI.<br>c. All surgeries were conducted in a dedicated aseptic condition. Rats were acclimatized for 7 days before surgery.<br>d. The injury model was chosen to mimic traumatic spinal cord injury in humans.
**Results**	10. For each experiment conducted, including independent replications, report:<br>a. Summary/descriptive statistics for each experimental group, with a measure of variability where applicable (e.g. mean and SD, or median and range).<br>b. If applicable, the effect size with a confidence interval.	**Results,** paragraph 3 at page 29 paragraph 2 and 3 at page 30<br>a. PBAE-A/N-treated animals exhibited significant functional recovery compared to the vehicle group (mock: 203 ± 15, treatment with PBAE-A/N plasmid: 156 ± 12; treatment with PBAE-A/N empty vector: 98 ± 17; mean ± SEM, n = 5 per group).<br>b. PBAE-A/N administration induced significant increase of BBB scores compared to the vehicle group.
