# Supplementary material for: PBAE nanoparticle-mediated delivery of ASCL1 and NGN2 genes for astroglia-to-neuron reprogramming to remodel glial scar for spinal cord injury repair
Source: Stem Cell Res Ther. 2026 Jul 4;17:335. doi: 10.1186/s13287-026-05159-2 (PMC13613702; doi:10.1186/s13287-026-05159-2)

Figure 1k

GFAP

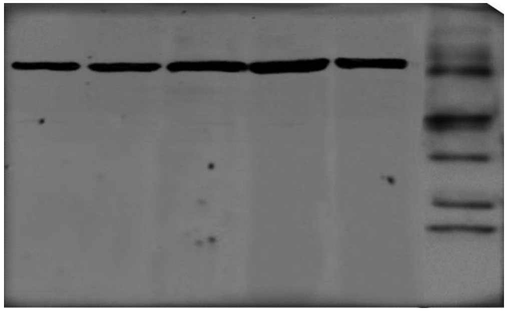

Brevican

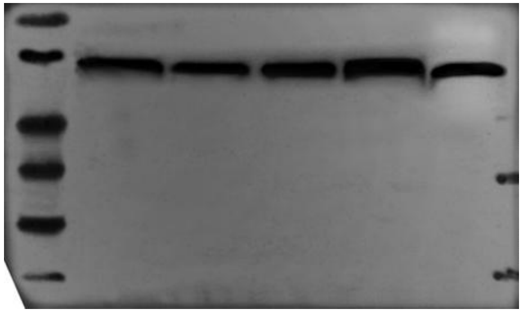

CSPG

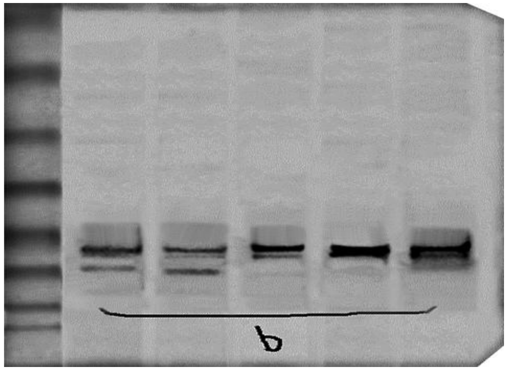

actin

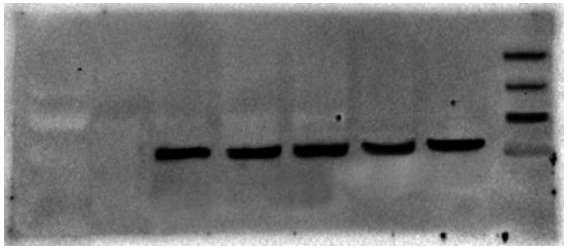

Figure 4C

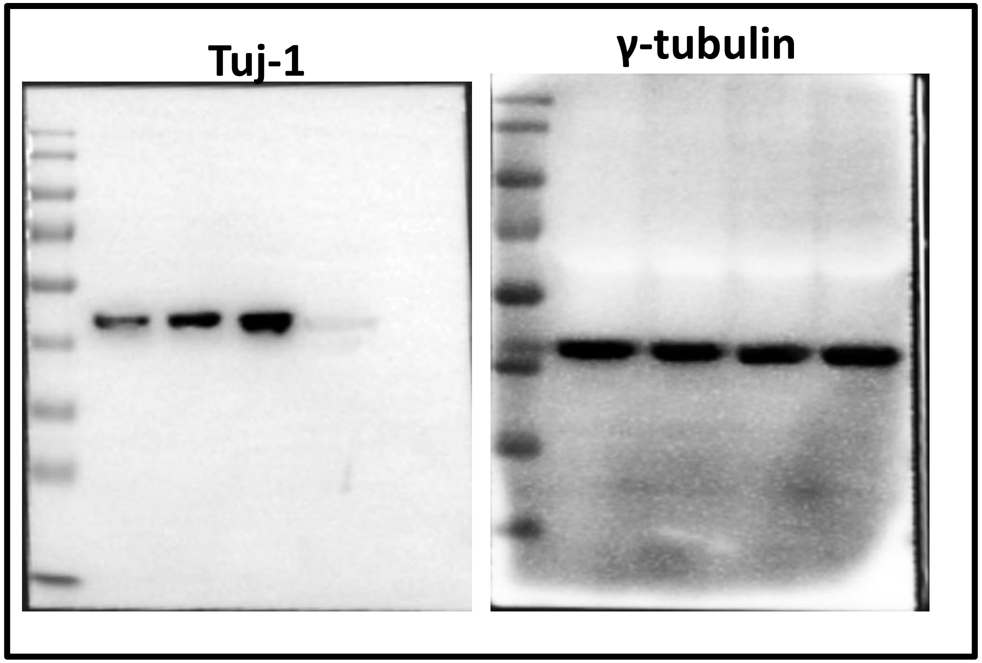

Figure 6j

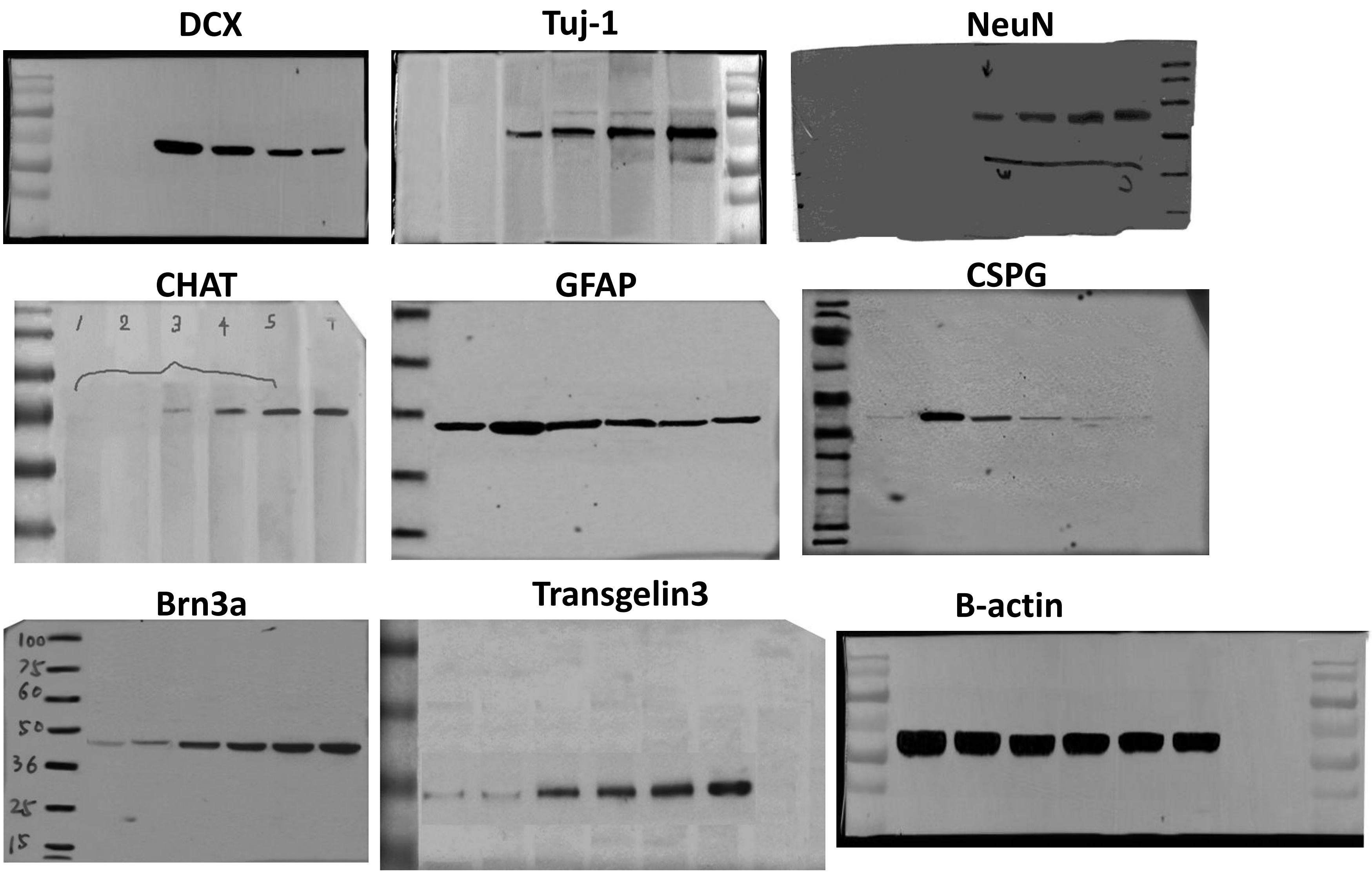
DCX
Tuj-1
NeuN
CHAT
1 2 3 4 5 T
GFAP
CSPG
Brn3a
100
75
60
50
36
25
15
Transgelin3
B-actin

Figure 8b

Synapsin

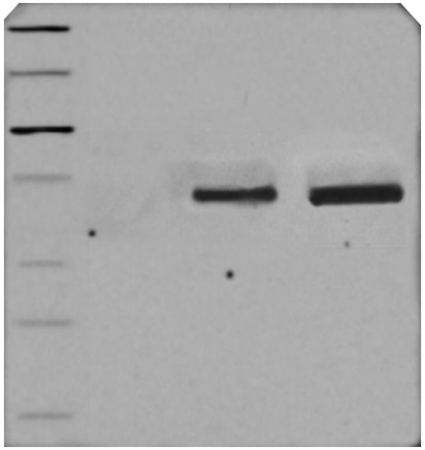

γ-tubulin

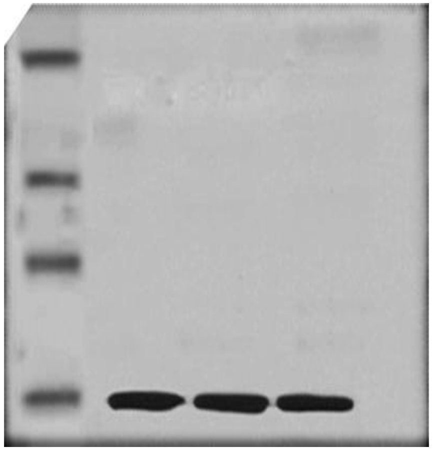

Figure 10e

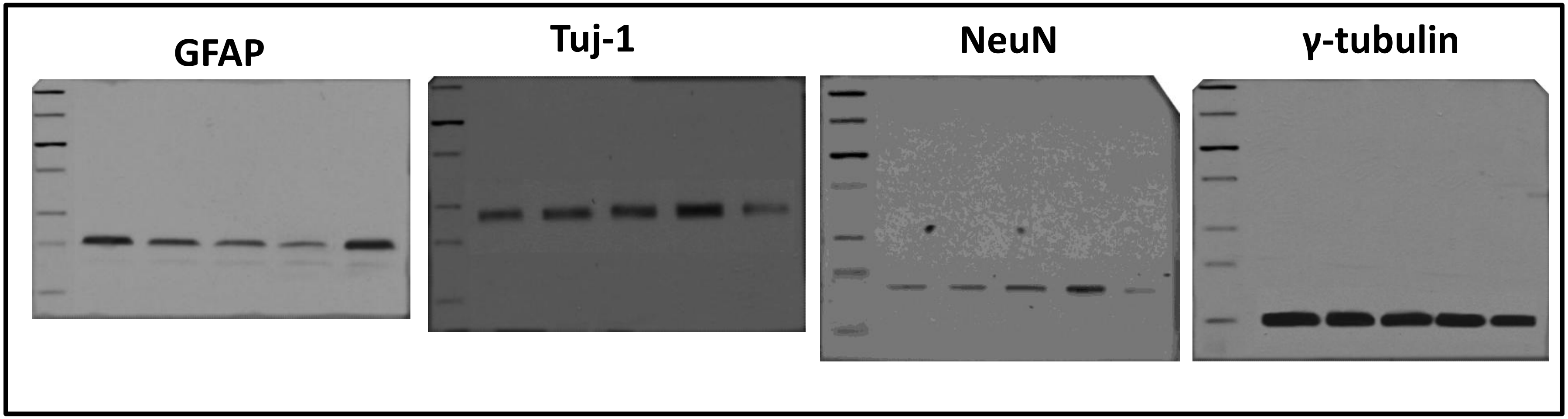

Figure 11a

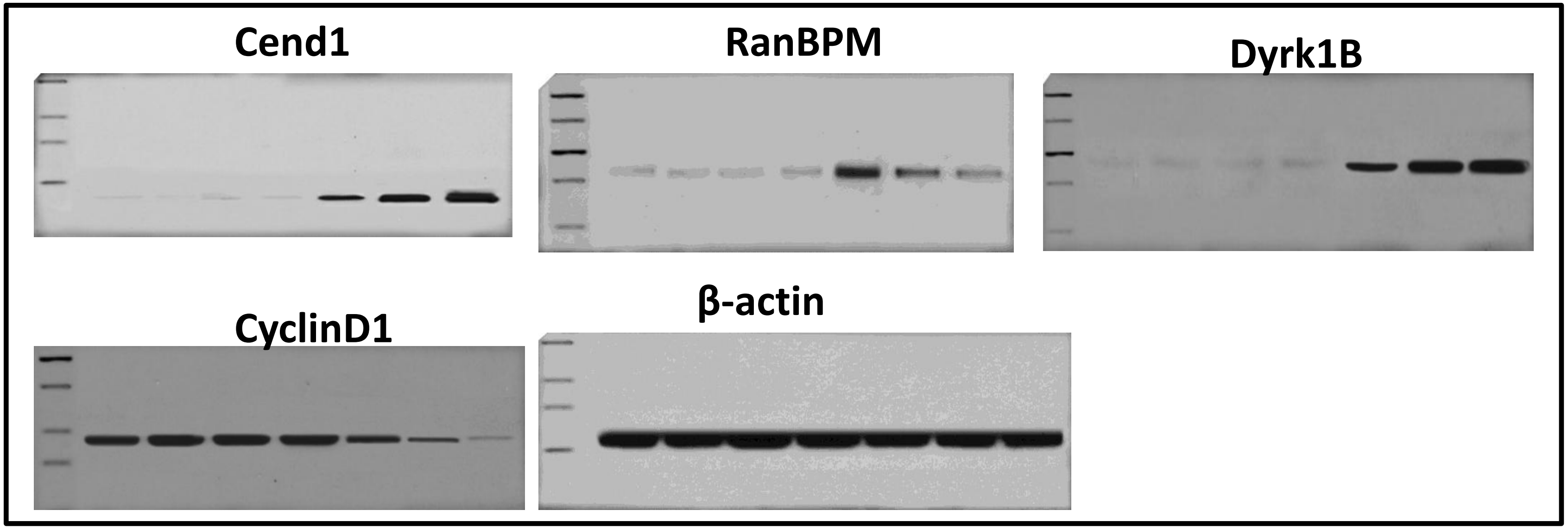

Figure 11g

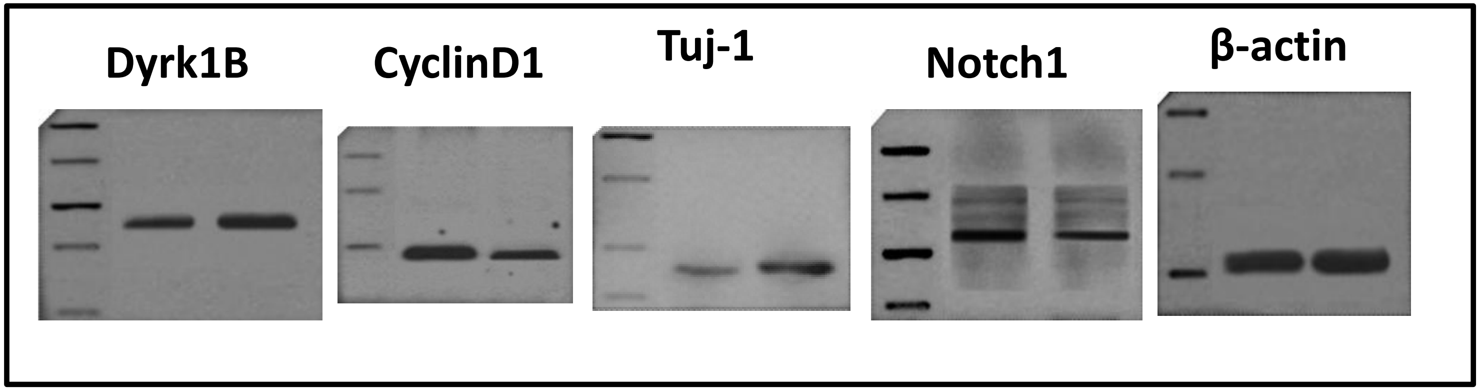

Figure 8h

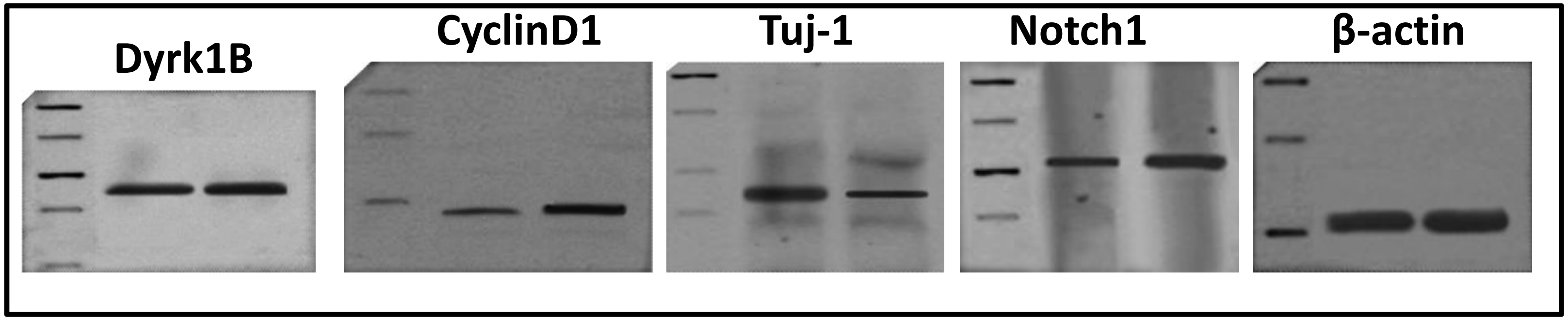

Supplement: Supplementary file 1 [file 13287_2026_5159_MOESM1_ESM.zip › original uncroped WB2.pdf]
