# Supplementary material for: PBAE nanoparticle-mediated delivery of ASCL1 and NGN2 genes for astroglia-to-neuron reprogramming to remodel glial scar for spinal cord injury repair
Source: Stem Cell Res Ther. 2026 Jul 4;17:335. doi: 10.1186/s13287-026-05159-2 (PMC13613702; doi:10.1186/s13287-026-05159-2)
Supplement: Supplementary file 1 [file 13287_2026_5159_MOESM1_ESM.zip › supplementary tables.pdf]

Table S1

Table S1 Primers used for reverse transcription polymerase chain reaction

Gene	Forward primer(F)	Reverse Primer(R)	Product size (bp)
**ASCL1**	5’-GCAACGCCAGATTACCTACC-3’	5’-GGTCGCCGCCTTATTTCTTC-3’	~144bp
**NGN2**	5’-CAGCGCAGTCAAGAAGACCA-3’	5’-GGCTGCTGTAGTAGGCGTTC-3’	~127bp
**DCX**	5'-AGAGCTGGAATGCGAACTGA-3'	5'-CCTCCTTCCCAACTGACCAC-3'	~150bp
**Tuj-1**	5'-CCAGCTGGTCTAAAGGCGAA-3'	5'-AAGGCATCTGGCATCTGGTC-3'	~109bp
**NeuN**	5'-CAGGACCATCAGCAAGAAAGC-3'	5'-TGCTCCTCAACTGGTTGTTCC-3'	~131bP
**CHAT**	5'-TGGGAGTCTGGAAGAACGAG-3'	5'-GATGCTGGGTCTGTCTGGTA-3'	~101bp
**Cend1**	5‘-GGAGAAGCTGAAGAGCGCTA-3’	5‘-GTCTTGGCTTCTCCAGCTTC-3’	~154bp
**Transgelin-3**	5‘-CCAGAGCAAGATGAGGACCA-3’	5‘-TTGTCCTTGGCATCCTTCTC-3’	~141bp
**Brn3a**	5‘-CGCAACCTCTACCGCAATAC-3’	5‘-GGTGTTGTGGGCTTCTTGTT-3’	~122bp
**GFAP**	5‘-CAGGAACGAACTGGCTCCTA-3’	5‘-GGAGAGGAAGGCTGAAGAAG-3’	~129bp
**CSPG**	5‘-TGAAGGAGCTGACCAACAAC-3’	5‘-GGCTTCTCCTTCATCAGCTC -3’	~108bp

Table S2

Table.S2 Antibody information

Antibody Name	Working Dilution	Diluent	Application	Cat. #	Manufacturer
Brevican	1:300/1:1000	2% BSA/PBS/TBST	ICC/WB	ab133616	Abcam
GFAP	1:200/1:1000	2% BSA/PBS/TBST	ICC/WB/IHC	2670	CST
CSPG	1:200/1:1000	2% BSA/PBS/TBST	ICC/WB	N8912	Sigma
Tuj-1	1:200/1:800	2% BSA/PBS/TBST	ICC/WB/IHC	4466	CST
CHAT	1:300/1:600	2% BSA/TBST	WB	Ab224267	Abcam
DCX	1:800	2% BSA/TBST	WB	13925-1-AP	Protintech
SYN	1:400/1:1000	2% BSA/PBS /TBST	ICC/WB	53059	CST
BrdU	1:300	2% BSA/PBS	ICC	5259	CST
NeuN	1:1000	2% BSA/TBST	WB	ABN78	Sigma
Brn3a	1:800	2% BSA/TBST	WB	ab81213	Abcam
Transgelin3	1:1000	2% BSA/TBST	WB	Ab14106	Abcam
Cend1	1:200/1:1000	2% BSA/PBS /TBST	ICC/WB	10R-10586	Fitzgerald
EDU	1:500	2% BSA/PBS	ICC	ab21901	Abcam
Cyclin D1	1:400/1:1000	2% BSA/TBST	ICC/WB	55506	CST
β-actin	1:2500	2% BSA/TBST	WB	81115-1-RR	Protintech
Dyrk1B	1:100/1:800	2% BSA/TBST	ICC/WB	2703	CST
RanBPM	1:200/1:1500	2% BSA/TBST	ICC/WB	OAEB00875	Aviva
Notch1	1:1000	2% BSA/TBST	WB	4380	CST
GFP	1:2000	2% BSA/PBS	WB	ab6556	Abcam
γ-tubulin	1:1500	2% BSA/TBST	WB	5886	CST
